# Identifying and evaluating clinical subtypes of Alzheimer’s disease in care electronic health records using unsupervised machine learning

**DOI:** 10.1186/s12911-021-01693-6

**Published:** 2021-12-08

**Authors:** Nonie Alexander, Daniel C. Alexander, Frederik Barkhof, Spiros Denaxas

**Affiliations:** 1 Institute of Health Informatics, University College London, London, UK; 2 Health Data Research UK, London, UK; 3 Centre for Medical Image Computing, Department of Computer Science, University College London, London, UK; 4 UCL Institute of Neurology, University College London, London, UK; 5 Alan Turing Institute, London, UK; 6 Department of Radiology and Nuclear Medicine, Amsterdam University Medical Centers, Amsterdam, The Netherlands

**Keywords:** Clustering, EHR, Alzheimer's disease, Subtyping, K-means

## Abstract

**Background:**

Alzheimer’s disease (AD) is a highly heterogeneous disease with diverse trajectories and outcomes observed in clinical populations. Understanding this heterogeneity can enable better treatment, prognosis and disease management. Studies to date have mainly used imaging or cognition data and have been limited in terms of data breadth and sample size. Here we examine the clinical heterogeneity of Alzheimer's disease patients using electronic health records (EHR) to identify and characterise disease subgroups using multiple clustering methods, identifying clusters which are clinically actionable.

**Methods:**

We identified AD patients in primary care EHR from the Clinical Practice Research Datalink (CPRD) using a previously validated rule-based phenotyping algorithm. We extracted and included a range of comorbidities, symptoms and demographic features as patient features. We evaluated four different clustering methods (k-means, kernel k-means, affinity propagation and latent class analysis) to cluster Alzheimer’s disease patients. We compared clusters on clinically relevant outcomes and evaluated each method using measures of cluster structure, stability, efficiency of outcome prediction and replicability in external data sets.

**Results:**

We identified 7,913 AD patients, with a mean age of 82 and 66.2% female. We included 21 features in our analysis. We observed 5, 2, 5 and 6 clusters in k-means, kernel k-means, affinity propagation and latent class analysis respectively. K-means was found to produce the most consistent results based on four evaluative measures. We discovered a consistent cluster found in three of the four methods composed of predominantly female, younger disease onset (43% between ages 42–73) diagnosed with depression and anxiety, with a quicker rate of progression compared to the average across other clusters.

**Conclusion:**

Each clustering approach produced substantially different clusters and K-Means performed the best out of the four methods based on the four evaluative criteria. However, the consistent appearance of one particular cluster across three of the four methods potentially suggests the presence of a distinct disease subtype that merits further exploration. Our study underlines the variability of the results obtained from different clustering approaches and the importance of systematically evaluating different approaches for identifying disease subtypes in complex EHR.

**Supplementary Information:**

The online version contains supplementary material available at 10.1186/s12911-021-01693-6.

## Introduction

### Background

Alzheimer's disease (AD) is a neurodegenerative disorder which affects 850,000 people in the UK with 95% of cases diagnosed in patients over 65. It is a biologically and clinically heterogeneous disease which varies in symptoms and rate of progression. In understanding this heterogeneity, it is important to examine not only the factors which vary but also the causes of that variation. For example, multiple factors affect the rate of progression, including education level [1], age of onset [2], comorbidities such as diabetes [3] and depression [4]. Thus, it is important not only to examine the heterogeneity of AD in patients but also to get a fuller picture of the health of patients with AD. Understanding this clinical heterogeneity is vital to tailoring treatment and providing accurate prognosis to patients, as well as the development of drugs.

The increasing availability of large medical datasets, combined with the application of machine learning methods offers new insights into different diseases. For example, clustering algorithms seek groups of patients more similar to each other than to patients in other groups [5] and thus can provide insight into the structure of disease heterogeneity. The division of patients into groups or subtypes can reveal new information and enhance predictive ability compared to examining an entire disease cohort as one homogeneous group [6]. Electronic health records (EHR) are routinely collected patient records provided by healthcare providers which contain information about symptoms and diagnoses, as well as lifestyle, drug prescriptions and demographic information. AD specifically is a highly heterogeneous disease which hampers diagnosis and management [7, 8]; clustering methods offer the potential to understand this heterogeneity better, but also require care in application to understand their utility in this context. The large number of patients and breadth of clinical information mean clustering methods offer new potential insight in the context of AD understanding.

### Related work

Other studies have used EHR to identify subtypes of AD. Xu et al. [9] used data from the multi-specialty urban academic medical center and hierarchical clustering and found 4 subtypes: one with patients who had higher proportions of cardiovascular disease, another subtype with higher prevalence of mental health conditions, a third subtype of multimorbid patients with later onset and a final subtype of patients who took anti-dementia drugs [9]. A second study used a representation learning model and hierarchical clustering on Mount Sinai Health System data and found 3 subtypes: an early onset mostly female cluster, a late onset cluster with mild neuropsychiatric symptoms and cerebrovascular disease and finally a cluster with mild to moderate dementia symptoms [10].

Other research subtyping AD focuses on two alternative types of data: cognitive tests [11–16] and brain anatomy studies using in-vivo brain scans or post mortem dissection [17–21]. Cognitive tests provide a quantitative score of the severity of memory loss and other signifiers of cognitive decline. Such studies have generally found a subtype of patients that have more severe memory problems with fewer other symptoms, and conversely other subtype (s) which have a larger array of different cognitive problems, yet their memory was less affected. The second approach uses brain scans either to measure atrophy patterns in different parts of the brain [17, 19, 22–24] or the buildup of molecular markers that are associated with AD [25–27]. Studies frequently do not always account for the progression of AD and it can be argued that rather than finding true AD subtypes they have in part identified different stages [12, 28]. Some studies have added a longitudinal dimension to their approach to clustering, in which the split between memory related symptoms and non memory related symptoms disappears [28–30]. There is currently little research on the difference of the clinical implications arising from each subtype in the UK population. For example, how patients in different subtypes respond to AD drugs, or how they interact with the healthcare system.

### This work

The aim of this work was to use EHR data [31] to discover and evaluate clinically meaningful subtypes of AD. The following steps outline the approach we took to achieve this:

1. We identified multiple different clinical cluster patterns of AD patients by applying four different clustering algorithms.
2. We evaluated each cluster method using three internal cluster validation metrics which measured cluster structure, cluster stability and cluster replicability. We did this in order to identify the best performing cluster method, and also to help identify clusters corresponding to true subtypes, as opposed to identifying artifacts of the clustering methods themselves. We further evaluated each approach in terms of its predictive value of two clinically relevant outcomes: firstly rate of decline in MMSE score, and secondly the length of time between diagnosis and the patient entering into assisted living.
3. Finally, we evaluated the consistency of the clusters found by comparing the results of the four cluster methods.

## Methods

### Data sources

We selected anonymized patient EHR from the Clinical Practice Research Datalink (CPRD) [32] which contains routinely collected data from general practices in the United Kingdom. CPRD contains longitudinal clinical information on patients, including demographic information, laboratory tests, diagnoses and symptoms encoded using the Read V2 controlled clinical terminology. Only patient records from primary care practices which met research data recording standards (known as Up To Standard and defined using CPRD algorithms examining patterns of data completeness and temporal gaps in recording) [32], and which produce data fit for use in research, were used. Data were extracted and phenotypes defined using the CALIBER data resource [33].

### Study population

The study period was between 1st of January 1997 and 30th June 2016. The start of the observation time was defined as the latest of:

1. When the patient joined the primary care practice.
2. When the primary care practice met research data recording standards [32].
3. 1st January 1997.

The end of the observation time was defined as the earliest of:

1. When the patient left the primary care practice.
2. When the patient died.
3. 1st June 2016.

We excluded patients with missing birth years or where the gender was not recorded.

We defined two cohorts of patients; an cohort of patients with AD and a secondary cohort of Unspecified Dementia (UD) patients as an external data set to test replicability. Patients were eligible for inclusion if the following conditions were met:

1. They were found to have either AD or UD diagnoses based on the CALIBER dementia phenotyping algorithm (details below).
2. The first recorded dementia diagnosis occurred after the age of 40.
3. There has been greater than or equal to one year of follow up before and after diagnosis.
4. There was at least one recorded symptom of AD such as memory loss or one recorded comorbidity.
5. Date of birth and gender information was available.

The AD cohort was split into a test set and a training set, in order to assess replicability. To compile the test set we selected 25% of practices at random and included all patients from those practices. Selecting patients from random practices is to mimic the effect of an external data set.

### Disease definition

We used a previously validated algorithm to identify AD patients, this algorithm defines an AD patient as having a diagnosis of AD and no further dementia subtype diagnosis. We identified the UD patients by the presence of a diagnosis of dementia but not a specific subtype, with no future diagnoses specifying a subtype. Phenotype definitions and associated Read codes can be found in Additional file 1: Table 1.

### Features for cluster analysis

To build a comprehensive clinical profile of the patient, three categories of variables were included in the analysis: symptoms, comorbidities and demographic and lifestyle factors.

Firstly, we conducted a systematic literature review of studies identifying symptoms of AD, and secondly, a review of diagnostic tests for AD. We searched the Web of Science Core Collection and Medline for papers. In order to identify symptoms of AD, we first searched using the terms “Alzheimer's Disease” AND “Symptoms” AND “prevalence”. We also conducted a systematic literature review to identify comorbidities associated with AD. We searched for the terms “Alzheimer's Disease” AND (“Disease” OR “Comorbidity”) AND (“risk” OR “progression” OR “protective”) and selected only systematic literature reviews.

The symptoms identified from our systematic literature review were agitation [34], anxiety [34], apathy [34], confusion [35], delirium [36], delusion [34], depression [34], difficulty walking [35], problems eating [34], fainting [37], falls [38], hallucinations [34], incontinence [39], language [35], memory [35], mood disorders [36], orientation [35], paranoia [39], seizure [37] and sleep issues [34]. We also carried out a systematic literature review to identify comorbidities associated with either an increased or reduced risk of AD. The following diseases were identified: atrial fibrillation [40], anxiety [40], hyperglycemia [41], hypercholesterolemia [42], rheumatoid arthritis [43], stroke [44], hearing loss [40], depression [40], kidney disease [45], heart failure [46], atherosclerosis [46] and cancer [47]. For demographic factors we included age of onset, gender, drinking status and smoking status.

We used predefined CALIBER phenotypes to define the features. If there was not a phenotype present, or if one did not exist for that disease, then a phenotype defined in a previous study was used (Additional file 1: Table 7). We excluded entries with missing dates. If the patient had no smoking information, they were defined as being a non-smoker. If a patient had no drinking status recorded they were defined as drinking status not specified, as that categorisation was part of the existing phenotype. The patient information was recorded in a matrix which had one row per patient and one column per variable. The presence or absence of a symptom or comorbidity is indicated with a 1 or 0 respectively. Categorical variables were one hot encoded—if one categorical variable had 5 different options recorded, that would become five columns with either a 1 or a 0.

Age at diagnosis was calculated by finding the difference between the date of AD or UD diagnosis and birth year. As some of the methods used only operate on categorical data, the age onset variable was divided into quintiles and each became a one hot encoded categorical variable. This is because our chosen dimensionality reduction method- MCA only takes categorical variables.

To ensure the presence of the symptom is related to AD, it was only recognised if two conditions were met: firstlly, if the symptom was recorded after the date of AD diagnosis and secondly if that symptom could not be explained by any other comorbidity (for example the symptom of depression and a previous diagnosis of depression). Hypercholesterolemia was removed because its prevalence in the CPRD dataset was too low. We selected only comorbidities that were present before diagnosis to ensure that they were not caused by AD.

### Outcomes

We defined five clinically relevant outcomes through examining the literature [13, 19] and consultations with clinicians. These were not included as features in the cluster definition but only used for validation purposes. The five outcomes were:

1. Length of time on Cholinesterase inhibitors: defined was length of time on cholinesterase inhibitors, as cholinesterase inhibitors (ChEI) are the most common type of drug prescribed for AD [48]. It is typically prescribed at the mild to moderate stage of AD [49] but is discontinued when the drugs are no longer beneficial to the patient. We identified the length of time between AD onset and the first GP appointment where no ChEIs were prescribed any longer. AD onset was used as a start date, as in the UK the first dementia drug prescription frequently does not occur in primary care [49]. The means and confidence intervals of this time difference were compared.
2. Time from diagnosis to assisted living: We compared time between diagnosis and assisted living by identifying clinical terms (Read codes) which indicated they were living in a care home or any change of address and compared rates between clusters by using a Kaplan-Meiers test.
3. Rate of dementia progression: We measured the rate of progression using the cognitive screening test Mini Mental State Exam (MMSE) and compared the decline in score year-on-year. Most patients have an MMSE score taken just before diagnosis and one sometime after [49]. We compared the rate of decline per year between clusters using means and confidence intervals.
4. Healthcare utilisation We investigated health care utilisation by comparing the frequency of in person consultations and the frequency of missed appointments between clusters.
5. Mortality Finally, all-cause mortality was compared between clusters using Kaplan–Meier curves.

### Statistical analysis

#### Clustering methods

We applied four clustering methods and derived multiple sets of clusters which we then compared and evaluated. We selected the following partitional methods as they all have different assumptions that influence the shape and type of identified clusters. The basic principles and assumptions of each of the four methods are outlined below.

##### K-means

K-means is a method that identifies k number of clusters through iteratively minimizing the distance between points and their assigned cluster means. We used multiple correspondence analysis (MCA) as a dimensionality reduction method [50] prior to clustering. MCA is the counterpart of principal component analysis,for categorical variables as most of the variables included are either categorical or binary. MCA lowers the number of dimensions of the data while representing the points in a geometrical space, thus transforming the data into a form where k-means can be applied. The number of components selected corresponds to the point at which the difference in variance explained by the component diminishes. We applied MCA to the entire dataset. We decided K through plotting elbow plots for the total sum of squares [5], silhouette coefficient [51] and Bayesian information criterion [52]. To find the best clustering solution the algorithm was repeated 100 times and the solution with the lowest total within cluster variance was identified.

##### Kernel K-means

Kernel k-means is similar to k-means but the data is transformed before using a kernel, this is to represent the data in a higher dimensional space to find non-linearly separable clusters that cannot be identified from normal k-means [5]. We used a Hamming distance kernel for categorical data [53]. We did not apply MCA to this data. K-means was then applied on the resulting matrix. The value for K and the best clustering solution was found using the same method as above.

##### Affinity propagation

Affinity propagation is a clustering method that identifies clusters by finding the best exemplar points in the dataset based on each pair of point’s similarity and each point’s availability to be an exemplar. Affinity propagation benefits from not needing a predetermined K and also finds non-linearly separable clusters [54]. First, we applied MCA as carried out in the k-means method then created a similarity matrix from the results. To find the best cluster solution we varied the preference which in turn resulted in different values for K. We then plotted the net similarity (the sum of the similarity from each point to its exemplar) for each result and determined the optimum cluster solution using an elbow plot.

##### Latent class analysis

Latent class analysis (LCA) presupposes that the distribution of the data is due to K number of underlying classes. It uses expectation maximisation to work out the probability of each point belonging to each of those classes [55]. We applied MCA to the original dataset and then ran LCA. It finds clusters that do not have equal variance and are not spherical. To identify the number of latent classes we ran LCA using two to eight different classes and the optimum class was identified using the lowest value of the Bayesian information criterion. Posterior probabilities were found for each patient for each class, the class with the greatest posterior probability is the one the patient was assigned to.

#### Cluster characterisation

We characterised each cluster by identifying features that were significantly different from the cohort as a whole, the clusters were then labeled with these features. For example, if a cluster had significantly higher depression and anxiety comorbidities it was labeled “anxiety and depression” cluster and if a cluster had memory problems but few other symptoms it was labeled as typical AD, as this is commonly used to describe these patients [56].

#### Evaluation measures

**Fig. 1:**
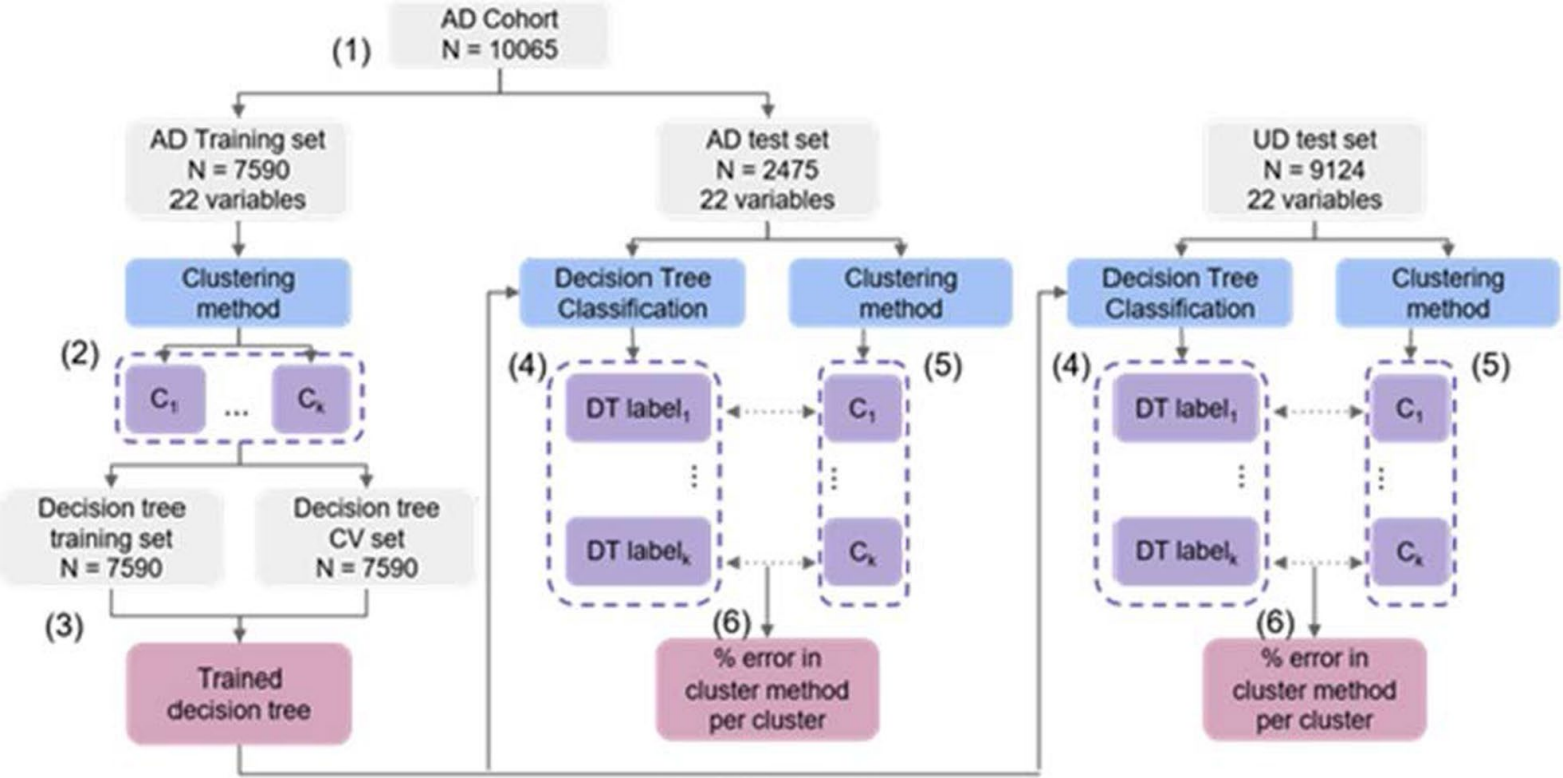
Reproducibility validation flow diagram showing how the AD cohort and UD cohort are used to validate the original AD clustering in different datasets: (1) splitting AD cohort into trial and test set, (2) using trial set to cluster patients using a cluster method, (3) split training set into a decision tree training and cross validation, then train a decision tree, (4) label test sets with trained decision tree (gold standard labels), (5) repeat cluster method, (6) find % discordance between decision tree labels and cluster labels to quantify reproducibility. *AD* Alzheimer's disease, *UD* unspecified dementia

We used five different metrics to evaluate the cluster results:

1. *Cluster structure* is a measure of how separated and distinct the clusters are. We used the silhouette coefficient to measure this [51]. The silhouette coefficient is a measure of how strongly each point associates with its assigned cluster relative to the next closest cluster. It returns a score between − 1 and 1 with higher scores indicating better cluster structure.
2. *Cluster stability* is a measure of how often the same cluster solution is found when repeating the same method on data from the same distribution. To measure this, we bootstrapped the sample 100 times, and repeated the cluster method on those samples. We then used a Jaccard coefficient to measure the overlap in the cluster results between each sample and the original. We took a mean of those scores, with possible values ranging from 0 to 1 (higher scores indicating more stable results). Scores of 0.75 were considered stable [57].
3. *Cluster replicability* is a measure of how well the results are found in external datasets. It is measured here through the % concordance of labels in the original and external datasets. We ran the cluster methods on two external data sets. The first is made up of patients from 25% of practices selected randomly, the second is a group of patients with unknown dementia. A decision tree was trained on the original data with cluster membership as outputs to label the external datasets with a gold standard. The cluster methods were then applied and the label concordance between the cluster method and decision tree indicated replicability (Fig. 1).
4. *Potential clinical utility* was defined as a measure of the predictive value of the cluster labels compared to the variables used in the clustering, if the cluster labels were found to have a higher predictive value than the variables, it shows that they are more clinically useful. We compared the predictive value of two outcomes: rate of progression using MMSE scores and time between diagnosis and assisted living. The first is examined by taking the adjusted R2 value of a linear model for each disease factor, and the second using cox proportional hazard ratio.
5. *Comparison of cluster solutions* was a method we used to examine if the clusters are robust and whether the four methods had identified hidden structures in the data rather than just artifacts. This was done visually using an alluvial plot.

## Results

### Cohort and data preprocessing

We identified 10,065 AD patients and 9124 UD patients from 11.3 million patient records that satisfied the cohort criteria, the dataset is 66% female (Table 1, Additional file 1: Fig. 1). This dataset was split into a training set of 7913 patients and a test set of 2152. As many of the symptoms had a very low prevalence, we grouped them into four broader symptom categories: memory, confusion, neuropsychological which is the occurrence of any of the following; aggression, agitation, anxiety, apathy, confusion, delirium, delusion, depression, hallucinations, sleep, eating, paranoia and mood disorders, and motor which is the occurrence of difficulty walking or orientation problems. MCA was applied and five principal components were identified.

**Table 1:** Patient demographics and symptom and comorbidity prevalence

	Percent	N
**Age**		
42–73	19.41	1954
74–78	20.04	2017
79–82	20.45	2058
83–87	20.24	2037
88+	19.87	2000
**Gender**		
Male	33.79	3401
Female	66.21	6664
**Smoking status**		
Non-Smoker	56.55	5692
Ex-smoker	32.94	3315
Current smoker	9.83	989
**Drinking Status**		
Non-drinker	27.79	2797
Ex-drinker	7.17	722
Occasional drinker	17.59	1770
Current drinker	1.19	120
**Symptom**		
Memory	78	7851
Confusion	24.8	2496
Neuropsychological symptoms	57.55	5792
Motor Symptoms	4.44	447
**Comorbidity**		
Anxiety	21.99	2213
Atherosclerosis	5.61	565
Atrial fibrillation	13.02	1310
Cancer	30.29	3049
Depression	27.06	2724
Diabetes	14.93	1503
Haemorrhagic stroke	1.91	192
Hearing loss	35.09	3532
Heart failure	7.43	748
Hyperglycaemia	1.42	143
Hypertension	60.55	6094
Kidney disease	29.01	2920
Rheumatoid arthritis	2.49	251

### Clustering results

#### K-means

**Fig. 2:**
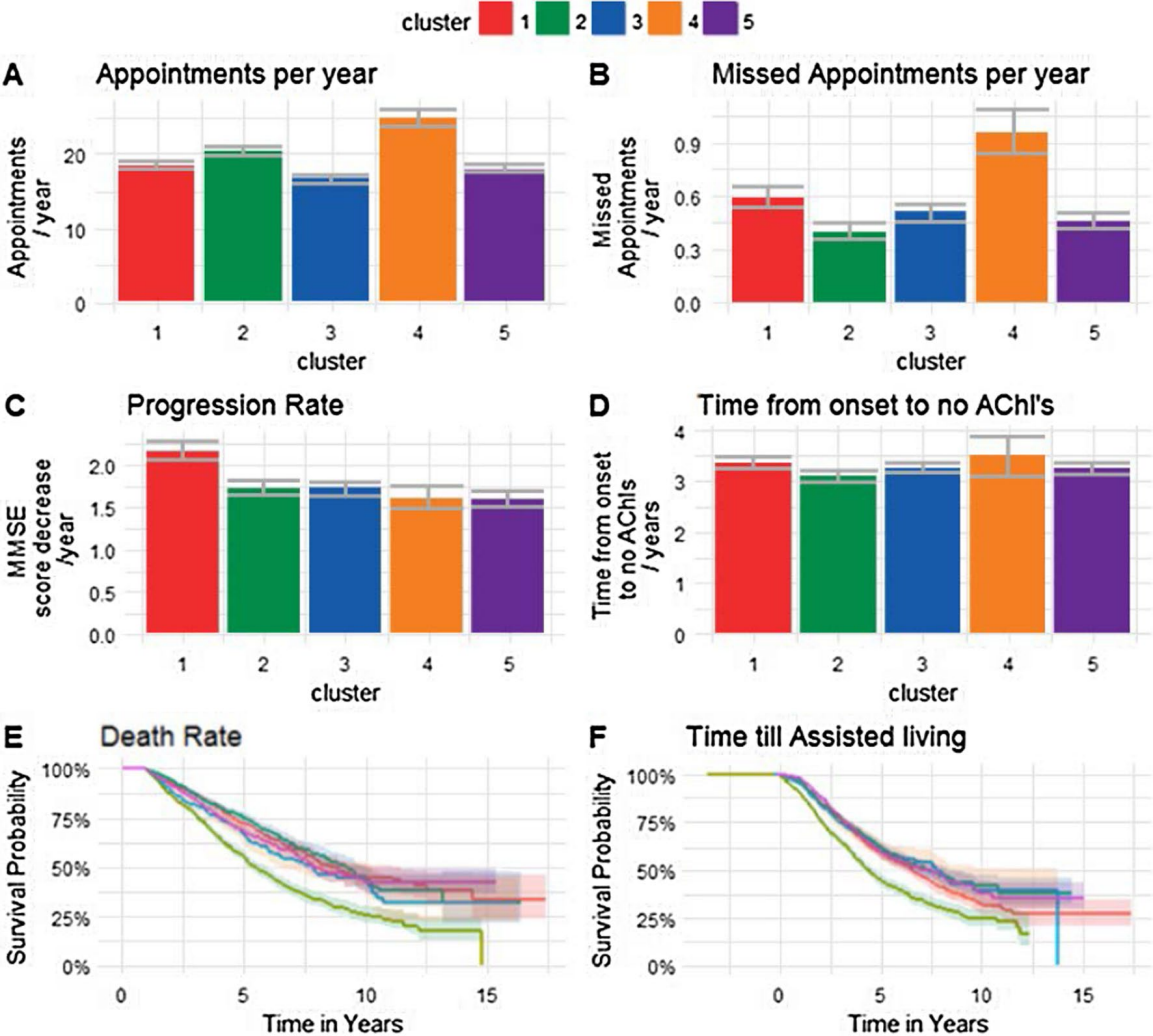
Outcomes of K-means clustering by cluster: **A** number of appointments per year post diagnosis with 5% confidence intervals, **B** number of missed appointments per year post diagnosis with 5% confidence intervals, **C** Progression rate based on decline in MMSE score per year with 5% confidence intervals, **D** time from onset of AD until AChls are stopped prescribed, with 5% confidence intervals, **E** Kaplan–Meier curve from diagnosis to death with log rank error, **F** Kaplan–Meier curve for time until the patient moves into assisted living with log rank error bars. *AD* Alzheimer's disease

**Fig. 3:**
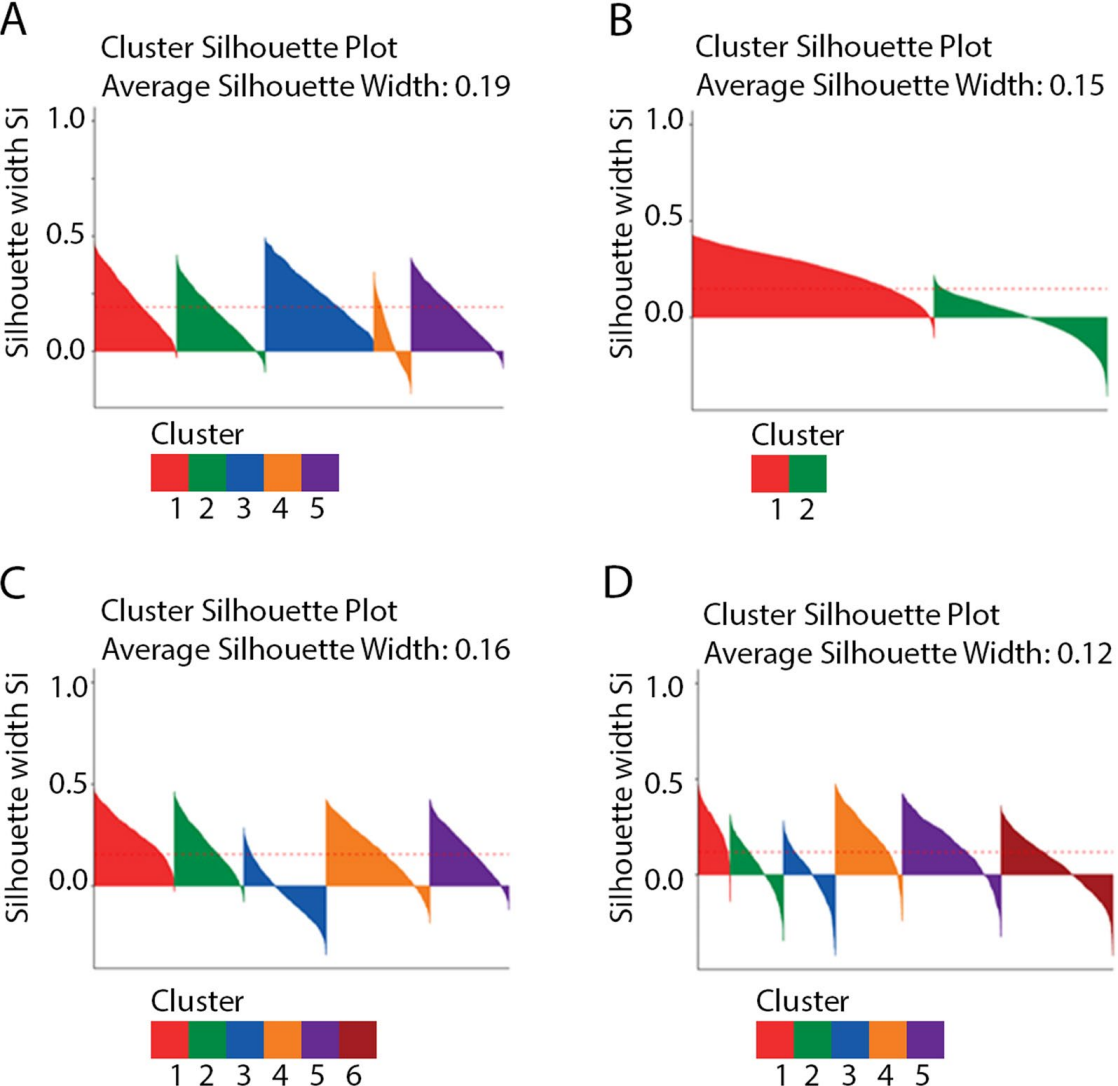
Silhouette plots of all samples results from: **A** k-means, **B** Kernel k-means, **C** Affinity propagation, **D** LCA. The dotted line represents the average silhouette score across all methods

The optimal number of clusters identified when using k-means was 5 (Additional file 1: Fig. 2): Anxiety and Depression, Early Onset and Smoking cluster, Non-typical AD cluster, Typical AD cluster, CVD cluster and a cluster of Men with Memory Problems and Cancer (Additional file 1: Fig. 3, Additional file 1: Table 2). The Anxiety and Depression cluster has the fastest progression based on MMSE score decline (Fig. 2). The silhouette score (Fig. 3A) was 0.19, showing weak cluster structure, the mean Jaccard coefficient was 0.78 indicating stable clusters (Additional file 1: Table 6a). There was a concordance between the cluster assignments found using the decision tree of 73% and 67% from the AD and UD data sets respectively (Additional file 1: Table 6b). These results indicate that this method is fairly robust at finding similar clusters in datasets which have greater variation of symptoms and dementia type compared to a dataset with purely AD patients (Additional file 1: Table 7).

#### Kernel K-means

The optimal number of clusters identified using Kernel k-means was 2 (Additional file 1: Fig. 4), these were a typical cluster and a comorbid cluster (Additional file 1: Fig. 5 and Additional file 1: Table 3). Despite being very stable clusters (Jaccard coefficient: 0.99) they showed weak cluster structure (silhouette score: 0.15, Fig. 3B) and were not found to be predictive of any outcomes (Additional file 1: Fig. 6). Using the decision tree, we found these clusters to be replicable in the test AD set (87% concordance) but the concordance drops in the UD dataset (73%).

**Fig. 4:**
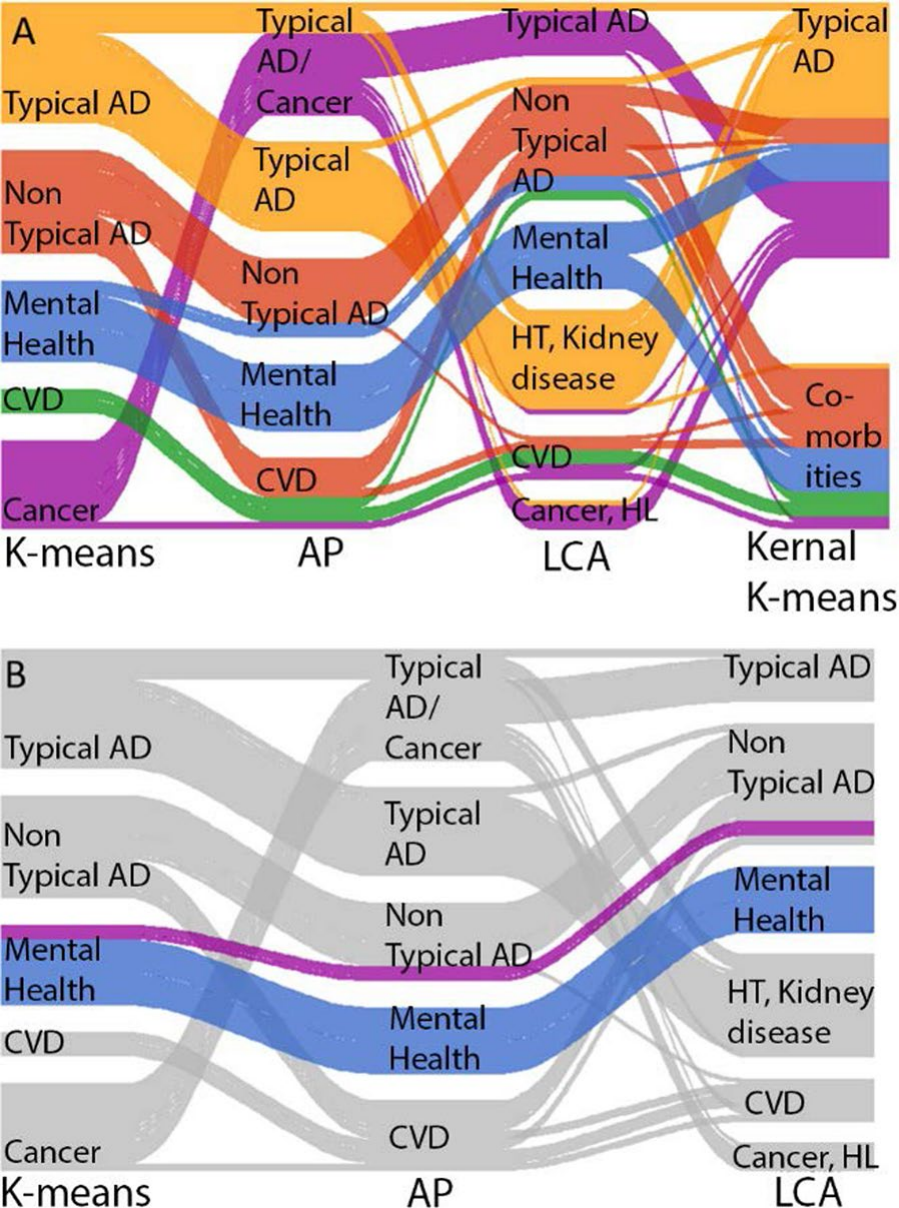
Alluvial plots showing patients transition to different clusters for each clustering method, **A** the colour represents the cluster in membership from k-means. **B** Highlights the anxiety and depression cluster for k-means, affinity propagation and LCA. *HT* hypertension, *HL* hearing loss, *AP* affinity propagation

#### Affinity propagation

Affinity Propagation identified 5 clusters (Additional file 1: Fig. 7): a Typical AD/Hyperglycaemia cluster, an Anxiety and Depression and Early Onset cluster, a CVD cluster, a Typical AD and Cancer cluster, and a Non-Typical AD cluster (Additional file 1: Fig. 8, Additional file 1: Table 4). Similar to k-means, an Anxiety and Depression cluster was found, however the Non-Typical AD cluster had the fastest rate of progression, this was followed by the Anxiety and Depression cluster (Additional file 1: Fig. 8). Affinity propagation had weak performance across all evaluation metrics especially cluster stability (silhouette score: 0.16, Fig. 3C, Jaccard's coefficient: 0.37, AD test concordance: 63%, UD test set concordance: 64%). However, cluster membership predicted time between diagnosis and assisted living better than any factor with the exception of confusion (Additional file 1: Fig. 9).

#### LCA

LCA identified 6 clusters (Additional file 1: Fig. 10): a Hearing Loss and Cancer cluster, a typical AD cluster, a CVD cluster, an Anxiety and Depression Early Onset and Smoking cluster, a Hypertension and Kidney Disease cluster and a Non-Typical AD cluster (Additional file 1: Fig. 11 and Additional file 1: Table 5).The Anxiety and Depression cluster was again found to have the quickest rate of progression (Additional file 1: Fig. 12). LCA had the worst cluster structure (silhouette score: 0.12, Fig. 3D) and weak stability (Jaccard's coefficient 0.67) it also performed badly when replicated in other datasets (AD test concordance: 54%, UD test set concordance: 65%). However these clusters did predict time between diagnosis and assisted living better than all disease factors with the exception of confusion. The clusters predicted time to death better than all disease factors with the exception of neuropsychological symptoms, memory and heart failure (Additional file 1: Table 7).

### Cluster method comparison

Here we compare the results from each cluster method to identify any consistent clusters or patterns across the different methods (Fig. 4).

#### Mental health, smoking and early onset cluster

This cluster appeared in the k-means, affinity propagation and LCA methods representing 1046 patients that had shared cluster features (Fig. 4B). This cluster had the fastest rate of progression in LCA and k-means and the second fastest in affinity propagation. As a cluster with these characteristics can be found with three different cluster methods with different assumptions it suggests that they have uncovered an underlying clustered structure to the data rather than an artefact of the methods.

#### Typical/cancer/hypertension cluster

Typical AD is a cluster found by all 4 methods and is defined by having high memory problems and low prevalence of other symptoms. In k-means two clusters exist with this pattern, though one also has a high prevalence of cancer. In kernel k-means this cluster also has a high prevalence of hypertension. In affinity propagation this pattern appears in two clusters: one which has high prevalence of cancer and one with high prevalence of hypertension. Half of the clusters found using the LCA method exhibit this pattern: two with high prevalence of cancer and hypertension again, and one with no significantly high comorbidities. The patients in the typical cluster in k-means are mostly in the typical cluster in affinity propagation, however some of those patients also form the hypertension cluster in LCA. Similarly the cluster characterised by having high levels of cancer in k-means are mostly in the Typical AD cluster in LCA. Both patients with cancer and hypertension share similar symptom profiles of high memory problems and infrequent other problems, but the clusters are mostly separated based on whether the patient had hypertension or cancer.

#### Non-typical / CVD

The Non-Typical cluster is labelled as such when there are high levels of all symptoms but memory loss. There is also a Non-Typical AD cluster found in LCA, k-means and affinity propagation; some patients that appear in the non-typical cluster with one method also appear in the CVD cluster in other methods. In the results generated by the k-means method, the CVD cluster has the smallest number of people. When using affinity propagation or LCA the CVD cluster is bigger as patients clustered in the non-typical AD cluster in k-means are classified as CVD in those two methods.

## Discussion

In this study we used UK primary care records to cluster AD patients using 4 different methods. K-means was the best performing method finding 4 clusters, and when we compared the results from all methods we found a consistent anxiety and depression cluster. Despite there being an abundance of research subtyping AD using cognitive tests, imaging data and genetics, this study is the one of the first to use EHR data for subtyping AD patients. This allowed us to include information from across the course of the disease rather than a cross section, allowed us to use both pre and post diagnosis data in the analysis and also allowed us to build a large clinical picture of the patient, including symptoms, comorbidities and prescriptions. This resulted in us clustering the entire clinical profile of patients with AD, expanding the subtype definition to encompass more aspects about the patient than just the disease alone. In this study we used UK primary care records to cluster AD patients, comparing four different clustering methods.

### Anxiety and depression cluster consistently found

An “Anxiety,Depression and Early-Onset” cluster was found in 3 of the 4 methods. This cluster was characterised by having high prevalence of depression, anxiety, current smokers and drinkers, with more than half of the cluster in the two earlier onset groups. They had a faster rate of progression—roughly 3 times as fast as the other clusters, which may be driven by the earlier onset of disease. A cluster with high mental health issues was also found in a previous study subtyping AD and EHR [9], and a subtype of early onset mostly female patients was found in another [10]. The latter study also suggested that in early onset cases prodromal signs of cognitive decline can be misdiagnosed as depression. A similar cluster was also found in a clustering analysis of patients with COPD, Pikoula et al. [58] suggesting that the combination of depression and anxiety as comorbidities of a disease can result in a clinical profile of those patients who have formed a distinct group separate from other patients with the same disease.

In all four methods a split between patients that had memory problems with few other symptoms and patients with neuropsychological problems, confusion and motor problems occurred. This divide appears in many previous studies such as one study finding two clusters, a memory predominant cluster and non-memory cluster [11], however that study found the non-memory cluster subjects were younger, whereas the corresponding cluster in this study was older at onset. Another study found 2 clusters of memory affected patients, 2 clusters of memory spared patients and a further 2 clusters which had either symptom [12]. This mirrors how we also found 3 or 4 clusters using 3 different methods which did not show any preference between memory or non-memory symptoms. One benefit of using EHR is that in the clusters that did not have a difference in symptoms, there were further distinctions in comorbidities. Studies looking at neurological-based clusters using brain scans and post mortem data also find a similar hippocampal [20] or entorhinal cortex [25] atrophy alongside a more diffuse, hippocampal sparing atrophy [17, 19], which correlates with greater memory loss and/or more diffuse symptoms. Studies using cross sectional data frequently have clusters separated by disease severity, which may be an indication the subtypes represent different stages in the disease. However, as we have used collapsed longitudinal data this study does not have that evaluative problem [12, 15, 16].

Typical AD was often associated with hypertension or cancer, suggesting a possible protective effect between these comorbidities and having a diffuse range of AD symptoms [46, 47]. It has been suggested that some cancers may be protective against dementia through the upregulation of proteins that increase cancer likelihood but prevent neurodegeneration [59].

A CVD cluster was found in three out of four of the methods employed, a finding seen in one other study subtyping AD using EHR [9]. Interestingly, patients with hypertension did not form part of the CVD cluster, with the two clusters having differing outcomes, suggesting that having hypertension or hypertension treatment results in a different AD profile than that of other CVD patients [60]. Non-Typical AD generally had a quicker rate of progression than typical AD patients, as well as a shorter time between diagnosis and assisted living, and greater healthcare utilisation.

Using EHR allowed us to collect a wide range of variables associated with AD as well allowing us to use a cohort that is representative of the UK [31]. The clusters found are relatable to features and outcomes that would be found in clinical practice. However, despite the benefits of EHR, there are some inherent issues with recording of variables and diagnoses. For example the dementia subtype may be initially recorded in primary care, changed in a memory clinic and not updated afterwards. Also, other important factors associated with AD such as family history are not systematically recorded in primary care EHR data so could not be included in the analysis. Although in this study we have measured clinical utility as an outcome, future research could involve clinical outcomes at either the data processing or clustering step as has been conducted in other studies [61–63]. Further research to verify the clusters using primary care data linked with data from a memory clinical will provide more reliable diagnoses and dementia specific variables.

The aim of our work was to compare clustering approaches and utilize them to generate hypotheses that can be further followed up with additional observational or interventional research. We used four different metrics to evaluate the different clustering methods, testing the cluster stability, structure, their clinical actionability and replicability. A comparison of method performance suggested that k-means offers the best solution as it performs the best in 2 out of 4 of the evaluation metrics. However, the cluster methods we used will return a solution irrespective of whether there is an underlying cluster structure, so it is necessary to test the solutions to ensure the results are not spurious. To do this, we compared the four methods to see if any clusters occurred consistently which gives confidence that those clusters are well defined patient subgroups with similar phenotypes.

## Conclusion

In this research we used four different clustering methods which produced inconsistent yet overlapping results and highlighted the need for systematic and robust evaluation. We observed a recurring cluster enriched for mental health disorders in three out of four clustering approaches. These findings highlight a clinically distinct cluster of AD patients that has been found in previous research [9] and can be a target for clinical intervention and further research. Future research should examine the best way to pick a cluster method and evaluate the results. An avenue for future research will be to investigate whether the clusters have genetic or neurophysiological differences. Future research should be conducted into the relationship between the factors in the cluster and AD, as to whether that relationship is causal.

## Supplementary Information

**Additional file 1:** **Figure S1.** CONSORT flow diagram of patient population from CPRD data showing how patients are excluded from the cohort. AD Alzheimer's Disease, UD Unspecified Dementia. **Figure S2.** Identification of optimum value of k with k-means run 100 times for each values of k where k = 2-14. Methods of measuring best value of k are a) Baysian Information Score, b) Silhouette score, c) Varience explained per cluster. **Figure S3.** Prevalence of each variable by cluster using k-means algorithm: A) Alzheimers symptoms, B) Comorbidies associated with AD (Alzheimer's Disease), C) Age, divided into quintiles, D) Gender by, E) Smoking status, F) Drinking Status. **Figure S4.** Identification of optimum value of k with kernel k-means run 100 times for each values of k where k = 2-8. Methods of measuring best value of k are a) Baysian Information Score, b) Silhouette score. **Figure S5.** Prevalence of each variable by cluster for kernel k-means: A) Alzheimers symptoms, B) Comorbidies associated with AD (Alzheimer's Disease), C) Age, divided into quintiles, D) Gender by, E) Smoking status, F) Drinking Status. **Figure S6.** Outcomes of kernel k-means clustering by cluster: a) number of appointments per year post diagnosis with 5% confidence intervals b) number of missed appointments per year post diagnosis with 5% confidence intervals, c) Progression rate based on decline in MMSE score per year with 5% confidence intervals, d) time from onset of AD until AChls are stopped prescribed, with 5% confidence intervals, e) Kaplan-Meier curve from diagnosis to death with log rank error, f) Kaplan-Meier curve for time until the patient moves into assisted living with log rank error bars. **Figure S7.** Identification of optimum value of k with affinity propergation run 100 times for each values of k 2-7 where the optimum value is found examining the net similarity to find the elbow in the plot. **Figure S8.** Prevalence of feature by cluster for affinity propagation: A) Alzheimers symptoms, B) Comorbidies associated with AD (Alzheimer's Disease), C) Age, divided into quintiles, D) Gender by, E) Smoking status, F) Drinking Status. **Figure S9.** Outcomes of affinity propagation clustering by cluster: a) number of appointments per year post diagnosis with 5% confidence intervals b) number of missed appointments per year post diagnosis with 5% confidence intervals, c) Progression rate based on decline in MMSE score per year with 5% confidence intervals, d) time from onset of AD until AChls are stopped prescribed, with 5% confidence intervals, e) Kaplan-Meier curve from diagnosis to death with log rank error, f) Kaplan-Meier curve for time until the patient moves into assisted living with log rank error bars. **Figure S10.** Identification of optimum value of k with LCA run 100 times for each values of k 2-8 where the optimum value is the minumum value for the baysian information criterion. **Figure S11.** Prevalence of feature by cluster for LCA: A) Alzheimers symptoms, B) Comorbidies associated with AD (Alzheimer's Disease), C) Age, divided into quintiles, D) Gender by, E) Smoking status, F) Drinking Status. **Figure S12.** Outcomes of LCA clustering by cluster: a) number of appointments per year post diagnosis with 5% confidence intervals b) number of missed appointments per year post diagnosis with 5% confidence intervals, c) Progression rate based on decline in MMSE score per year with 5% confidence intervals, d) time from onset of AD until AChls are stopped prescribed, with 5% confidence intervals, e) Kaplan-Meier curve from diagnosis to death with log rank error, f) Kaplan-Meier curve for time until the patient moves into assisted living with log rank error bars. **Table S1.** Sources and references for variables included in the analysis and phenotypes used to extract the variables from the EHR data. **Table S2.** K-means results % prevalence of each variable by cluster. **Table S3.** Kernal K-means results % prevalence of each variable by cluster. **Table S4.** Affinity propagation results % prevalence of each variable by cluster. **Table S5.** LCA results % prevalence of each variable by cluster. **Table S6a.** Cluster stability based on jaccard score of cluster membership overlap based on k-means from bootstrapped data run 100 times. **Table S6b.** Concordance between test set cluster membership determined by cluster method and cluster membership based on a decision tree trained on the original cluster results for each cluster method for the AD and UD test data sets. **Table S7a.** R squared values and adjusted r squared value for each variable based on a linear regression predicting decline in mmse score. **Table S7b.** Results of the cox proportional hazard ratio time till death of each variable included in the cluster analysis and cluster membership. **Table S7c.** Cox proportional hazard ratio time till assisted living of each variable included in the cluster analysis and cluster membership. **codelists.** codelists used for defining phenotypes for diseases and symptoms.

## Data Availability

The data that support the findings of this study are available from Clinical Practice Research Datalink (CPRD; www.cprd.com) but restrictions apply to the availability of these data, which were used under license for the current study, and so are not publicly available. For re-using these data, an application must be made directly to CPRD.

## Abbreviations

AD: Alzheimer's disease
ChEI: Cholinesterase Inhibitors
CPRD: Clinical Practice Research Link
EHR: Electronic Health Records
LCA: Latent Class Analysis
MCA: Multiple correspondence analysis
MMSE: Mini Mental State Exam
UD: Unspecified dementia
