# Supplementary material for: Identifying and evaluating clinical subtypes of Alzheimer’s disease in care electronic health records using unsupervised machine learning
Source: BMC Med Inform Decis Mak. 2021 Dec 8;21:343. doi: 10.1186/s12911-021-01693-6 (PMC8653614; doi:10.1186/s12911-021-01693-6)
Supplement: Supplementary file 1 — Additional file 1: Figure S1. CONSORT flow diagram of patient population from CPRD data showing how patients are excluded from the cohort. AD Alzheimer's Disease, UD Unspecified Dementia. Figure S2. Identification of optimum value of k with k-means run 100 times for each values of k where k = 2-14. Methods of measuring best value of k are a) Baysian Information Score, b) Silhouette score, c) Varience explained per cluster. Figure S3. Prevalence of each variable by cluster using k-means algorithm: A) Alzheimers symptoms, B) Comorbidies associated with AD (Alzheimer's Disease), C) Age, divided into quintiles, D) Gender by, E) Smoking status, F) Drinking Status. Figure S4. Identification of optimum value of k with kernel k-means run 100 times for each values of k where k = 2-8. Methods of measuring best value of k are a) Baysian Information Score, b) Silhouette score. Figure S5. Prevalence of each variable by cluster for kernel k-means: A) Alzheimers symptoms, B) Comorbidies associated with AD (Alzheimer's Disease), C) Age, divided into quintiles, D) Gender by, E) Smoking status, F) Drinking Status. Figure S6. Outcomes of kernel k-means clustering by cluster: a) number of appointments per year post diagnosis with 5% confidence intervals b) number of missed appointments per year post diagnosis with 5% confidence intervals, c) Progression rate based on decline in MMSE score per year with 5% confidence intervals, d) time from onset of AD until AChls are stopped prescribed, with 5% confidence intervals, e) Kaplan-Meier curve from diagnosis to death with log rank error, f) Kaplan-Meier curve for time until the patient moves into assisted living with log rank error bars. Figure S7. Identification of optimum value of k with affinity propergation run 100 times for each values of k 2-7 where the optimum value is found examining the net similarity to find the elbow in the plot. Figure S8. Prevalence of feature by cluster for affinity propagation: A) Alzheimers symptoms, B) Comor [file 12911_2021_1693_MOESM1_ESM.docx]

# Supplementary Figures

#### *
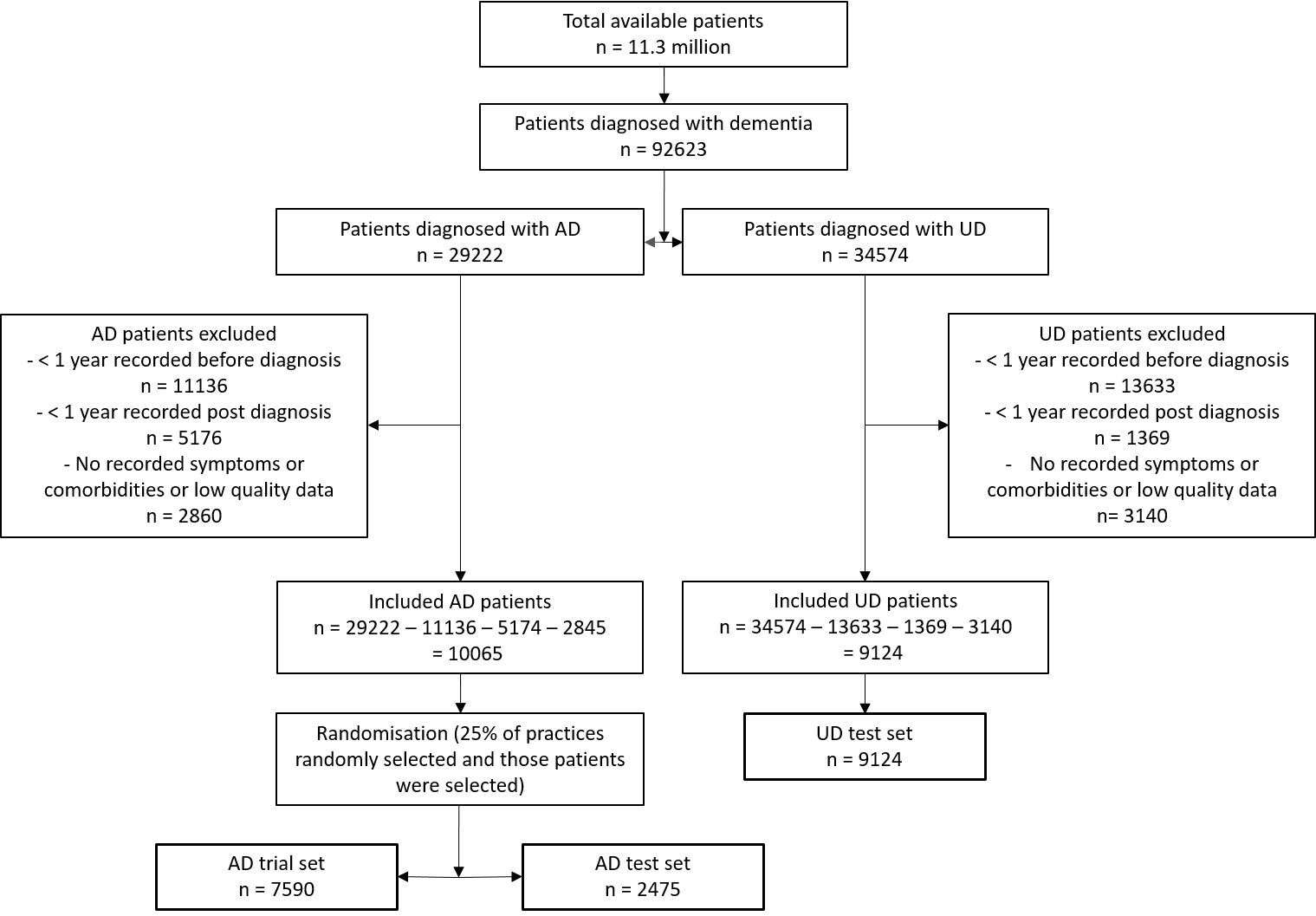
*

### Supplementary Figure 1

CONSORT flow diagram of patient population from CPRD data showing how patients are excluded from the cohort

AD Alzheimer's Disease, UD Unspecified Dementia


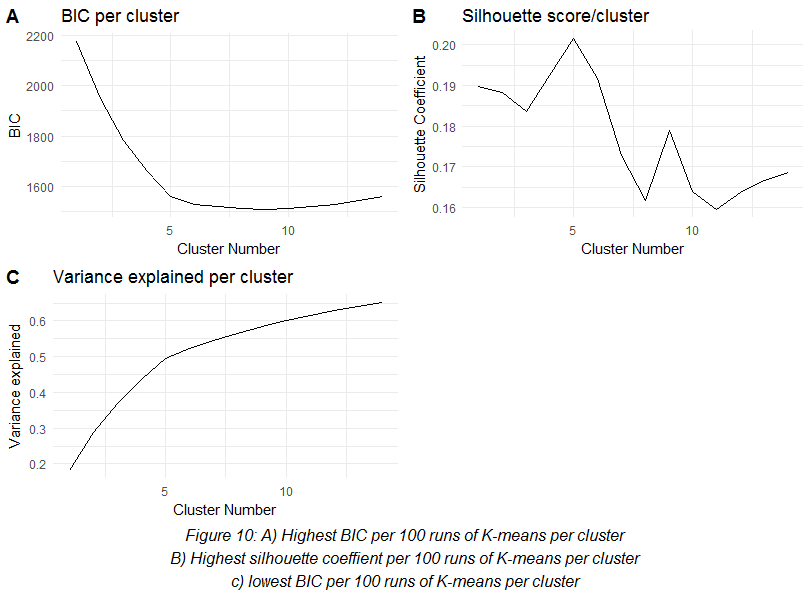


### Supplementary Figure 2

Idenfication of optimum value of k with k-means run 100 times for each values of k where k = 2-14. Methods of measuring best value of k are a) Baysian Information Score, b) Silhouette score, c) Varience explained per cluster.

###
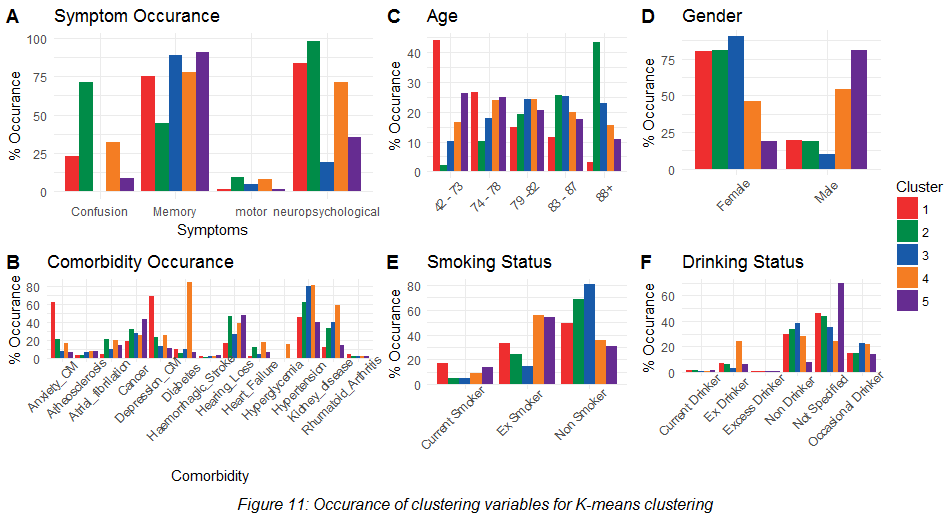
Supplementary Figure 3

Prevalence of each variable by cluster using k-means algorithm: A) Alzheimers symptoms, B) Comorbidies associated with AD (Alzheimer's Disease), C) Age, divided into quintiles, D) Gender by, E) Smoking status, F) Drinking Status


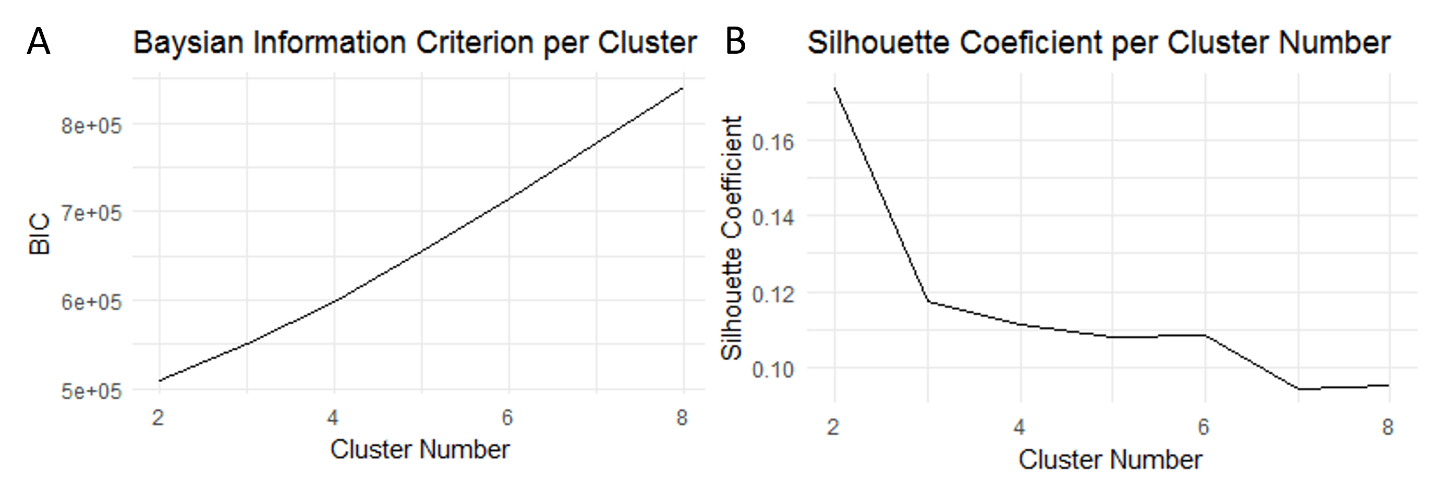


### Supplementary Figure 4

Identification of optimum value of k with kernel k-means run 100 times for each values of k where k = 2-8. Methods of measuring best value of k are a) Baysian Information Score, b) Silhouette score,

###
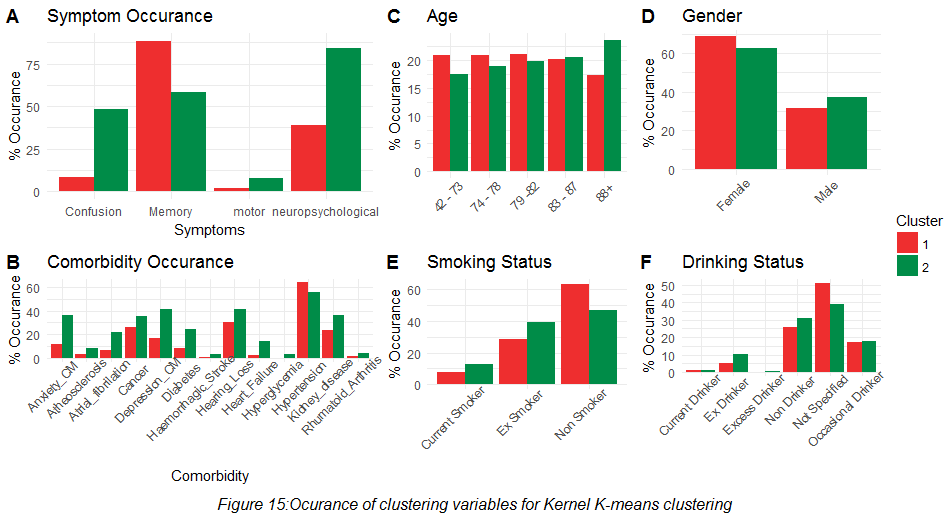


### Supplementary Figure 5

Prevalence of each variable by cluster for kernel k-means: A) Alzheimers symptoms, B) Comorbidies associated with AD (Alzheimer's Disease), C) Age, divided into quintiles, D) Gender by, E) Smoking status, F) Drinking Status


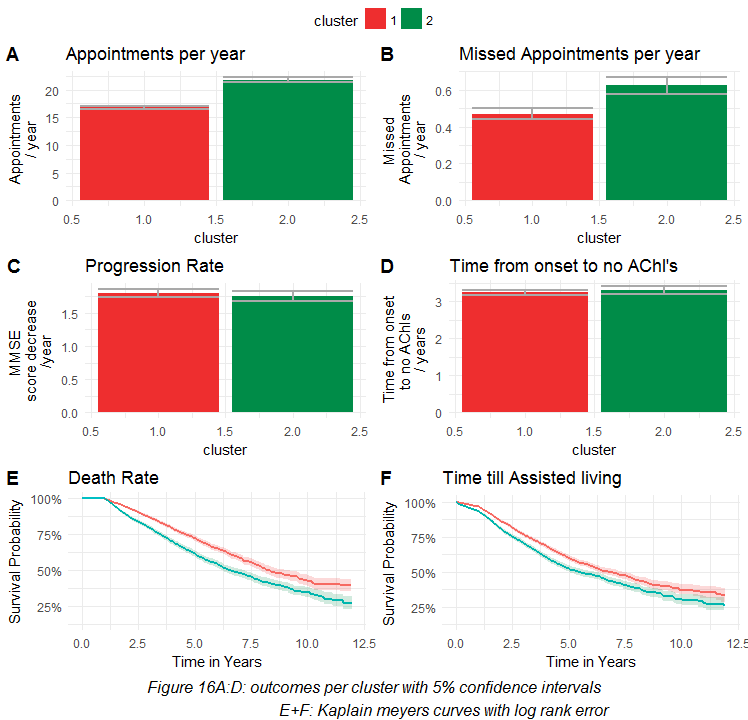


### Supplementary Figure 6

Outcomes of kernel k-means clustering by cluster: a) number of appointments per year post diagnosis with 5% confidence intervals b) number of missed appointments per year post diagnosis with 5% confidence intervals, c) Progression rate based on decline in MMSE score per year with 5% confidence intervals, d) time from onset of AD until AChls are stopped prescribed, with 5% confidence intervals, e) Kaplan-Meier curve from diagnosis to death with log rank error, f) Kaplan-Meier curve for time until the patient moves into assisted living with log rank error bars.


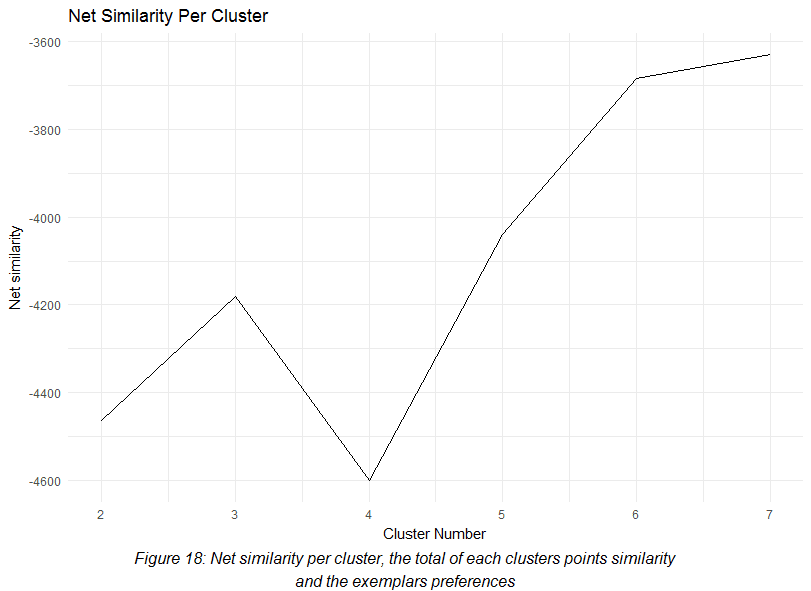


### Supplementary Figure 7

Identification of optimum value of k with affinity propergation run 100 times for each values of k 2-7 where the optimum value is found examining the net similarity to find the elbow in the plot

###
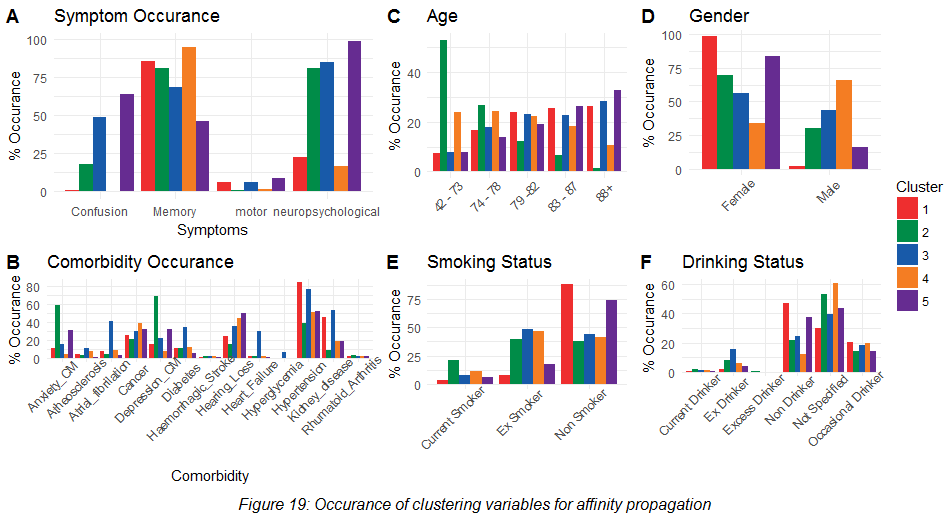


### Supplementary Figure 8

Prevalence of feature by cluster for affinity propagation: A) Alzheimers symptoms, B) Comorbidies associated with AD (Alzheimer's Disease), C) Age, divided into quintiles, D) Gender by, E) Smoking status, F) Drinking Status


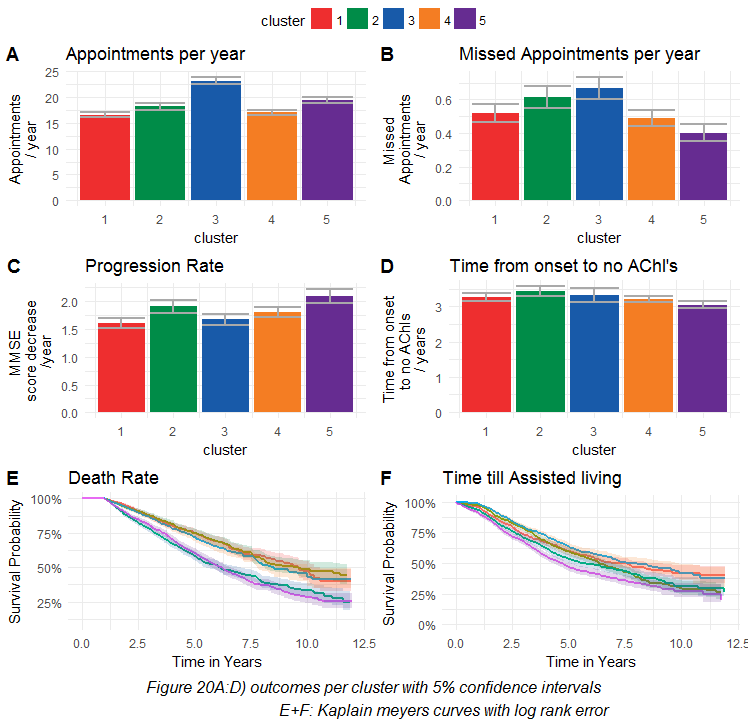


### Supplementary Figure 9

Outcomes of affinity propagation clustering by cluster: a) number of appointments per year post diagnosis with 5% confidence intervals b) number of missed appointments per year post diagnosis with 5% confidence intervals, c) Progression rate based on decline in MMSE score per year with 5% confidence intervals, d) time from onset of AD until AChls are stopped prescribed, with 5% confidence intervals, e) Kaplan-Meier curve from diagnosis to death with log rank error, f) Kaplan-Meier curve for time until the patient moves into assisted living with log rank error bars.


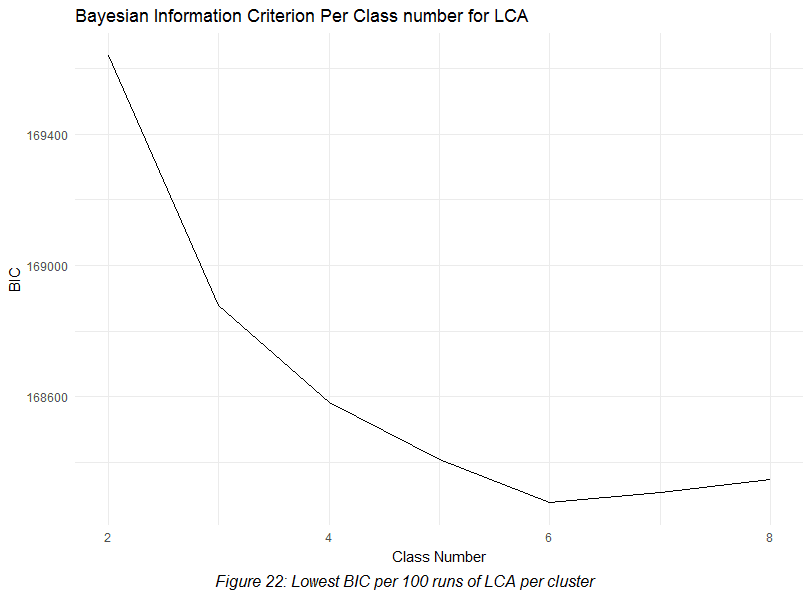


### Supplementary Figure 10

Identification of optimum value of k with LCA run 100 times for each values of k 2-8 where the optimum value is the minumum value for the baysian information criterion

###
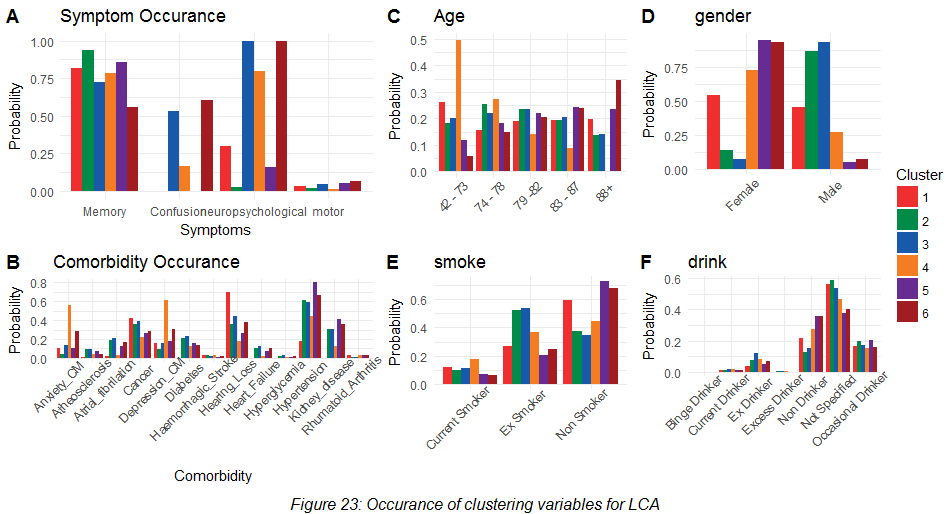


### Supplementary Figure 11

Prevalence of feature by cluster for LCA: A) Alzheimers symptoms, B) Comorbidies associated with AD (Alzheimer's Disease), C) Age, divided into quintiles, D) Gender by, E) Smoking status, F) Drinking Status


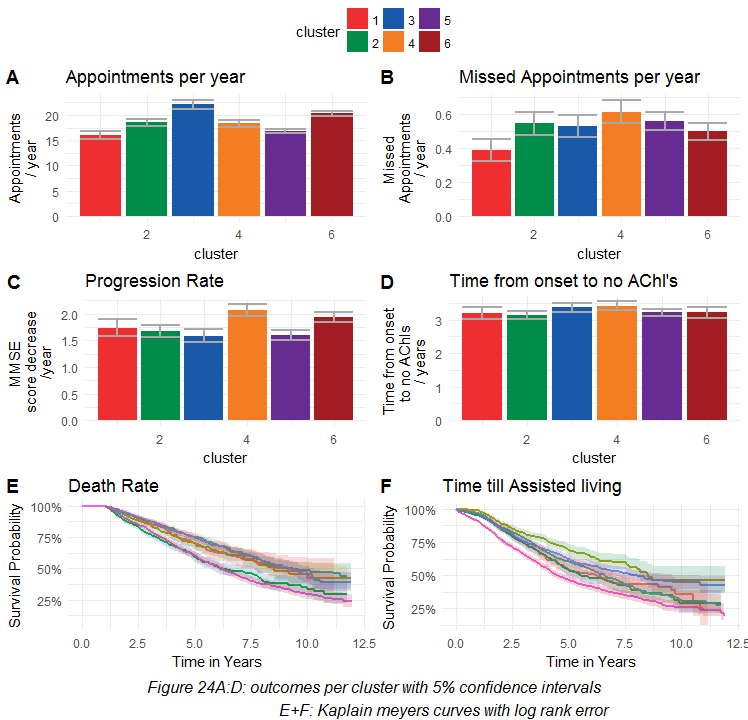


### Supplementary Figure 12

Outcomes of LCA clustering by cluster: a) number of appointments per year post diagnosis with 5% confidence intervals b) number of missed appointments per year post diagnosis with 5% confidence intervals, c) Progression rate based on decline in MMSE score per year with 5% confidence intervals, d) time from onset of AD until AChls are stopped prescribed, with 5% confidence intervals, e) Kaplan-Meier curve from diagnosis to death with log rank error, f) Kaplan-Meier curve for time until the patient moves into assisted living with log rank error bars.

#

# Supplementary Tables

| **Symptom type** | **Symptom** | **Source** | **Phenotyping Method** |
| --- | --- | --- | --- |
| **Neuropsychological** | delusion | [Prevalence of Neuropsychiatric Symptoms in Dementia and Mild Cognitive Impairment](https://jamanetwork.com/journals/jama/fullarticle/195320?utm_source=TrendMD&utm_medium=cpc&utm_campaign=J_Am_Med_TrendMD_1) | Prodromal dementia phenotype |
|  | hallucinations | [Prevalence of Neuropsychiatric Symptoms in Dementia and Mild Cognitive Impairment](https://jamanetwork.com/journals/jama/fullarticle/195320?utm_source=TrendMD&utm_medium=cpc&utm_campaign=J_Am_Med_TrendMD_1) | Prodromal dementia phenotype |
|  | agitation | [Prevalence of Neuropsychiatric Symptoms in Dementia and Mild Cognitive Impairment](https://jamanetwork.com/journals/jama/fullarticle/195320?utm_source=TrendMD&utm_medium=cpc&utm_campaign=J_Am_Med_TrendMD_1) | Prodromal dementia phenotype |
|  | aggression | [Prevalence of Neuropsychiatric Symptoms in Dementia and Mild Cognitive Impairment](https://jamanetwork.com/journals/jama/fullarticle/195320?utm_source=TrendMD&utm_medium=cpc&utm_campaign=J_Am_Med_TrendMD_1) | Prodromal dementia phenotype |
|  | depression | [Prevalence of Neuropsychiatric Symptoms in Dementia and Mild Cognitive Impairment](https://jamanetwork.com/journals/jama/fullarticle/195320?utm_source=TrendMD&utm_medium=cpc&utm_campaign=J_Am_Med_TrendMD_1) | - |
|  | anxiety | [Prevalence of Neuropsychiatric Symptoms in Dementia and Mild Cognitive Impairment](https://jamanetwork.com/journals/jama/fullarticle/195320?utm_source=TrendMD&utm_medium=cpc&utm_campaign=J_Am_Med_TrendMD_1) | [caliber phenotype](https://github.com/spiros/datalab/blob/master/projects/CALIBER/phenotypes_2016/5_Mental_and_behavioral_disorders/Anxiety/anxiety_cprd/cal_anxiety.sql) |
|  | apathy | [Prevalence of Neuropsychiatric Symptoms in Dementia and Mild Cognitive Impairment](https://jamanetwork.com/journals/jama/fullarticle/195320?utm_source=TrendMD&utm_medium=cpc&utm_campaign=J_Am_Med_TrendMD_1) | me |
|  | sleep | [Prevalence of Neuropsychiatric Symptoms in Dementia and Mild Cognitive Impairment](https://jamanetwork.com/journals/jama/fullarticle/195320?utm_source=TrendMD&utm_medium=cpc&utm_campaign=J_Am_Med_TrendMD_1) | Prodromal dementia phenotype |
|  | eating | [Prevalence of Neuropsychiatric Symptoms in Dementia and Mild Cognitive Impairment](https://jamanetwork.com/journals/jama/fullarticle/195320?utm_source=TrendMD&utm_medium=cpc&utm_campaign=J_Am_Med_TrendMD_1) | Prodromal dementia phenotype |
|  | suspicion/paranoia | [Clinical Spectrum, Risk Factors, and Behavioral Abnormalities among Dementia Subtypes in a North Indian Population: A Hospital-Based Study](https://www.karger.com/Article/Pdf/478978) | Prodromal dementia phenotype |
|  | delirium | [Delirium episodes during the course of clinically diagnosed Alzheimer's disease.](https://www.ncbi.nlm.nih.gov/pmc/articles/PMC2608571/) | Prodromal dementia phenotype |
|  | mood disorders | [Delirium episodes during the course of clinically diagnosed Alzheimer's disease.](https://www.ncbi.nlm.nih.gov/pmc/articles/PMC2608571/) | Prodromal dementia phenotype |
|  |  |  |  |
| **Motor** | falls | [The diagnosis, burden and prognosis of dementia: A record-linkage cohort study in England](http://journals.plos.org/plosone/article?id=10.1371/journal.pone.0199026) | [caliber phenotype](https://github.com/spiros/datalab/blob/master/projects/CALIBER/phenotypes_2016/5_Mental_and_behavioral_disorders/Dementia/dementia_fall_cprd/cal_dementia_fall_cprd_1.sql) |
|  | orientation | [A New Rating Scale for Alzheimer’s Disease](https://ajp.psychiatryonline.org/doi/pdf/10.1176/ajp.141.11.1356) | Prodromal dementia phenotype |
|  | difficulty walking | [A New Rating Scale for Alzheimer’s Disease](https://ajp.psychiatryonline.org/doi/pdf/10.1176/ajp.141.11.1356) | Prodromal dementia phenotype |
|  |  |  |  |
| **Cognitive** | memory | [A New Rating Scale for Alzheimer’s Disease](https://ajp.psychiatryonline.org/doi/pdf/10.1176/ajp.141.11.1356) | [caliber phenotype](https://github.com/spiros/datalab/blob/master/projects/CALIBER/phenotypes_2016/5_Mental_and_behavioral_disorders/Dementia/dementia_memory_cprd/cal_dementia_memory_cprd_1.sql) |
|  | language | [A New Rating Scale for Alzheimer’s Disease](https://ajp.psychiatryonline.org/doi/pdf/10.1176/ajp.141.11.1356) | Prodromal dementia phenotype |
|  | confusion | [A New Rating Scale for Alzheimer’s Disease](https://ajp.psychiatryonline.org/doi/pdf/10.1176/ajp.141.11.1356) | [caliber phenotype](https://github.com/spiros/datalab/blob/master/projects/CALIBER/phenotypes_2016/5_Mental_and_behavioral_disorders/Dementia/dementia_confusion_cprd/cal_dementia_confusion_cprd_1.sql) |
|  |  |  |  |
| **Autonomic** | incontinence | [Clinical Spectrum, Risk Factors, and Behavioral Abnormalities among Dementia Subtypes in a North Indian Population: A Hospital-Based Study](https://www.karger.com/Article/Pdf/478978) | [First Diagnosis and Management of Incontinence in Older People with and without Dementia in Primary Care: A Cohort Study Using The Health Improvement Network Primary Care Database](http://journals.plos.org/plosmedicine/article?id=10.1371/journal.pmed.1001505#s3) |
|  | seizure | [Seizures in Alzheimer Disease](https://jamanetwork.com/journals/jamaneurology/fullarticle/797760) | [Implementing near real-time vaccine safety surveillance using the Clinical Practice Research Datalink (CPRD)](https://www.sciencedirect.com/science/article/pii/S0264410X17312392?via%3Dihub#s0120) |
|  | fainting | [Seizures in Alzheimer Disease](https://jamanetwork.com/journals/jamaneurology/fullarticle/797760) | me |

### Supplementary table 1

Sources and references for variables included in the analysis and phenotypes used to extract the variables from the EHR data.

| **Symptom/**  **comorbidity** | **1** | **2** | **3** | **4** | **5** |
| --- | --- | --- | --- | --- | --- |
| Anxiety | 62.11 | 20.98 | 7.90 | 16.47 | 6.02 |
| Atherosclerosis | 3.14 | 3.41 | 6.27 | 8.16 | 8.13 |
| Atrial fibrillation | 3.86 | 20.79 | 9.58 | 20.41 | 14.85 |
| Cancer | 19.04 | 32.20 | 27.69 | 26.09 | 43.27 |
| Confusion | 22.71 | 71.04 | 0.05 | 32.36 | 8.60 |
| Depression | 69.24 | 22.99 | 13.03 | 25.51 | 10.53 |
| Diabetes | 9.75 | 5.67 | 10.02 | 84.26 | 6.43 |
| Haemorrhagic Stroke | 2.03 | 1.40 | 1.58 | 1.60 | 2.81 |
| Hearing Loss | 16.88 | 46.59 | 26.95 | 38.92 | 48.42 |
| Heart Failure | 2.23 | 12.56 | 4.49 | 17.93 | 6.43 |
| Hyperglycemia | 0.00 | 0.00 | 0.00 | 15.74 | 0.00 |
| Hypertension | 45.94 | 61.89 | 80.60 | 81.49 | 40.18 |
| Kidney disease | 12.30 | 33.72 | 39.88 | 58.89 | 14.56 |
| Memory | 74.93 | 44.15 | 89.09 | 77.70 | 90.94 |
| motor | 1.31 | 9.45 | 4.39 | 7.58 | 1.23 |
| neuropsychological | 83.51 | 98.35 | 18.85 | 71.43 | 35.50 |
| Rheumatoid Arthritis | 3.99 | 2.32 | 2.27 | 2.33 | 1.64 |
| **Age** |  |  |  |  |  |
| 42 - 73 | 43.98 | 2.01 | 10.27 | 16.47 | 26.14 |
| 74 - 78 | 26.51 | 10.12 | 17.77 | 23.76 | 24.97 |
| 79 -82 | 14.73 | 19.27 | 24.23 | 24.34 | 20.64 |
| 83 - 87 | 11.58 | 25.49 | 25.02 | 19.83 | 17.43 |
| 88+ | 3.21 | 43.11 | 22.70 | 15.60 | 10.82 |
| **Gender** |  |  |  |  |  |
| Female | 80.17 | 81.28 | 90.18 | 45.92 | 19.01 |
| Male | 19.83 | 18.72 | 9.82 | 54.08 | 80.99 |
| **Smoking Status** |  |  |  |  |  |
| Current Smoker | 17.41 | 5.24 | 4.79 | 9.04 | 14.09 |
| Ex-Smoker | 32.92 | 24.15 | 14.31 | 55.39 | 54.44 |
| Non-Smoker | 48.95 | 68.84 | 80.90 | 35.57 | 31.11 |
| **Drinking Status** |  |  |  |  |  |
| Current Drinker | 1.57 | 1.16 | 0.84 | 0.58 | 1.52 |
| Ex-Drinker | 7.26 | 6.10 | 2.86 | 24.34 | 6.32 |
| Excess Drinker | 0.46 | NA | NA | NA | 0.47 |
| non-Drinker | 29.52 | 33.48 | 38.55 | 28.57 | 7.72 |
| Not Specified | 46.01 | 44.21 | 35.14 | 24.34 | 69.59 |
| Occasional Drinker | 15.18 | 15.06 | 22.61 | 22.16 | 14.39 |

### Supplementary table 2

K-means results % prevalence of each variable by cluster

| **Symptom/**  **comorbidity** | 1 | 2 |
| --- | --- | --- |
| Anxiety | 11.77 | 36.28 |
| Atherosclerosis | 3.34 | 8.79 |
| Atrial fibrillation | 6.57 | 22.03 |
| Cancer | 26.30 | 35.87 |
| Confusion | 8.13 | 48.10 |
| Depression | 16.52 | 41.81 |
| Diabetes | 8.29 | 24.21 |
| Haemorrhagic Stroke | 1.04 | 3.13 |
| Hearing Loss | 30.64 | 41.31 |
| Heart Failure | 2.67 | 14.10 |
| Hyperglycemia | 0.23 | 3.10 |
| Hypertension | 63.90 | 55.88 |
| Kidney disease | 23.66 | 36.50 |
| Memory | 88.54 | 58.25 |
| motor | 1.92 | 7.96 |
| neuropsychological | 38.66 | 83.98 |
| Rheumatoid Arthritis | 1.27 | 4.20 |
| **Age** |  |  |
| 42 - 73 | 20.88 | 17.35 |
| 74 - 78 | 20.85 | 18.90 |
| 79 -82 | 20.92 | 19.79 |
| 83 - 87 | 20.11 | 20.42 |
| 88+ | 17.24 | 23.55 |
| **Gender** |  |  |
| Female | 68.66 | 62.77 |
| Male | 31.34 | 37.23 |
| **Smoking Status** |  |  |
| Current Smoker | 7.84 | 12.80 |
| Ex-Smoker | 28.33 | 39.38 |
| non-Smoker | 63.47 | 46.87 |
| **Drinking Status** |  |  |
| Current Drinker | 1.04 | 1.39 |
| Ex-Drinker | 5.08 | 10.08 |
| Excess Drinker | 0.09 | 0.35 |
| non-Drinker | 25.55 | 30.91 |
| Not Specified | 50.99 | 39.19 |
| Occasional Drinker | 17.24 | 18.08 |

### Supplementary table 3

Kernal K-means results % prevalence of each variable by cluster

| **Symptom/**  **comorbidity** | 1 | 2 | 3 | 4 | 5 |
| --- | --- | --- | --- | --- | --- |
| Anxiety | 10.73 | 59.46 | 15.96 | 3.84 | 30.73 |
| Atherosclerosis | 4.89 | 3.39 | 10.7 | 7.32 | 0.76 |
| Atrial fibrillation | 7.33 | 4.34 | 41.02 | 8.42 | 3.32 |
| Cancer | 25.12 | 21.45 | 29.92 | 38.68 | 32.66 |
| Confusion | 0.54 | 18.06 | 48.34 | 0 | 63.53 |
| Depression | 15.55 | 68.93 | 21.68 | 8.05 | 32.66 |
| Diabetes | 11.47 | 10.73 | 34.24 | 12 | 5.88 |
| Haemorrhagic Stroke | 1.22 | 2.44 | 1.99 | 2.58 | 1.18 |
| Hearing Loss | 24.37 | 15.69 | 35.37 | 44.95 | 49.76 |
| Heart Failure | 2.51 | 1.89 | 29.45 | 2.16 | 1.31 |
| Hyperglycemia | 0.07 | 0.32 | 6.05 | 0.32 | 0.42 |
| Hypertension | 84.18 | 38.56 | 76.8 | 50.68 | 51.83 |
| Kidney disease | 45.28 | 8.75 | 53.26 | 18.32 | 19.03 |
| Memory | 85.47 | 80.99 | 67.95 | 94.26 | 45.88 |
| Motor problems | 5.91 | 0.87 | 6.25 | 1.21 | 8.44 |
| neuropsychological | 22.47 | 80.76 | 84.77 | 16.42 | 98.69 |
| Rheumatoid Arthritis | 2.44 | 3.71 | 2.19 | 1.89 | 2.56 |
| **Age** |  |  |  |  |  |
| 42 - 73 | 7.47 | 53.08 | 7.91 | 24.05 | 7.89 |
| 74 - 78 | 16.84 | 26.58 | 17.89 | 24.53 | 13.91 |
| 79 -82 | 23.83 | 12.30 | 23.14 | 22.21 | 19.03 |
| 83 - 87 | 25.66 | 6.70 | 22.81 | 18.37 | 26.37 |
| 88+ | 26.21 | 1.34 | 28.26 | 10.84 | 32.80 |
| **Gender** |  |  |  |  |  |
| Female | 98.30 | 69.48 | 56.05 | 33.79 | 83.81 |
| Male | 1.70 | 30.52 | 43.95 | 66.21 | 16.19 |
| **Smoking Status** |  |  |  |  |  |
| Current Smoker | 3.67 | 21.37 | 7.65 | 11.63 | 6.30 |
| Ex-Smoker | 8.08 | 39.67 | 48.60 | 46.63 | 18.06 |
| Non-Smoker | 88.26 | 37.93 | 43.75 | 41.53 | 73.63 |
| **Drinking Status** |  |  |  |  |  |
| Current Drinker | 0.95 | 1.97 | 1.46 | 1.11 | 0.55 |
| Ex-Drinker | 1.97 | 7.97 | 15.49 | 6.37 | 4.15 |
| Excess Drinker | NA | 0.71 | NA | 0.21 | 0.14 |
| Non-Drinker | 46.91 | 21.69 | 24.73 | 12.32 | 37.16 |
| Not Specified | 29.74 | 53.31 | 39.83 | 60.53 | 43.88 |
| Occasional Drinker | 20.43 | 14.35 | 18.48 | 19.47 | 14.12 |

### Supplementary table 4

Affinity propagation results % prevalence of each variable by cluster

| **Symptom/**  **comorbidity** | class 1: | class 2: | class 3: | class 4: | class 5: | class 6: |
| --- | --- | --- | --- | --- | --- | --- |
| Anxiety | 0.11 | 0.04 | 0.14 | 0.55 | 0.10 | 0.28 |
| Atherosclerosis | 0.01 | 0.09 | 0.09 | 0.04 | 0.07 | 0.04 |
| Atrial fibrillation | 0.02 | 0.19 | 0.21 | 0.03 | 0.12 | 0.17 |
| Cancer | 0.42 | 0.36 | 0.40 | 0.22 | 0.27 | 0.28 |
| Confusion | 0.00 | 0.00 | 0.53 | 0.16 | 0.00 | 0.61 |
| Depression | 0.16 | 0.10 | 0.16 | 0.62 | 0.18 | 0.31 |
| Diabetes | 0.00 | 0.21 | 0.24 | 0.12 | 0.16 | 0.14 |
| Haemorrhagic Stroke | 0.03 | 0.03 | 0.02 | 0.03 | 0.01 | 0.01 |
| Hearing Loss | 0.70 | 0.36 | 0.44 | 0.18 | 0.26 | 0.38 |
| Heart Failure | 0.00 | 0.10 | 0.13 | 0.02 | 0.07 | 0.10 |
| Hyperglycemia | 0.00 | 0.00 | 0.00 | 0.00 | 0.00 | 0.00 |
| Hypertension | 0.00 | 0.02 | 0.03 | 0.01 | 0.01 | 0.02 |
| Kidney disease | 0.18 | 0.61 | 0.60 | 0.44 | 0.80 | 0.67 |
| Memory | 0.00 | 0.31 | 0.30 | 0.12 | 0.42 | 0.35 |
| motor | 0.82 | 0.94 | 0.72 | 0.78 | 0.86 | 0.56 |
| neuropsychological | 0.04 | 0.02 | 0.05 | 0.01 | 0.05 | 0.07 |
| Rheumatoid Arthritis | 0.30 | 0.02 | 1.00 | 0.80 | 0.16 | 1.00 |
| **Age** | 0.03 | 0.01 | 0.01 | 0.03 | 0.03 | 0.03 |
| 42 - 73 |  |  |  |  |  |  |
| 74 - 78 | 0.26 | 0.18 | 0.20 | 0.50 | 0.12 | 0.06 |
| 79 -82 | 0.16 | 0.25 | 0.22 | 0.27 | 0.18 | 0.15 |
| 83 - 87 | 0.19 | 0.23 | 0.23 | 0.14 | 0.22 | 0.21 |
| 88+ | 0.20 | 0.19 | 0.21 | 0.09 | 0.24 | 0.24 |
| **Gender** | 0.20 | 0.14 | 0.14 | 0.00 | 0.24 | 0.35 |
| Female |  |  |  |  |  |  |
| Male | 0.46 | 0.86 | 0.93 | 0.27 | 0.05 | 0.07 |
| **Smoking Status** | 0.54 | 0.14 | 0.07 | 0.73 | 0.95 | 0.93 |
| Current Smoker |  |  |  |  |  |  |
| Ex-Smoker | 0.59 | 0.37 | 0.35 | 0.45 | 0.73 | 0.68 |
| non-Smoker | 0.27 | 0.52 | 0.54 | 0.37 | 0.20 | 0.25 |
| **Drinking Status** | 0.12 | 0.10 | 0.11 | 0.18 | 0.07 | 0.06 |
| Current Drinker |  |  |  |  |  |  |
| Ex-Drinker | 0.22 | 0.12 | 0.15 | 0.28 | 0.36 | 0.36 |
| Excess Drinker | 0.04 | 0.08 | 0.12 | 0.08 | 0.05 | 0.07 |
| Non-Drinker | 0.16 | 0.20 | 0.17 | 0.15 | 0.21 | 0.16 |
| Not Specified | 0.01 | 0.01 | 0.02 | 0.01 | 0.01 | 0.01 |
| Occasional Drinker | 0.00 | 0.00 | 0.00 | 0.01 | 0.00 | 0.00 |
| Occasional Drinker | 0.56 | 0.59 | 0.54 | 0.47 | 0.37 | 0.40 |

### Supplementary table 5

LCA results % prevalence of each variable by cluster

###

###

| Method | Mean Jaccard's Index |
| --- | --- |
| K-Means | 0.78 |
| Kernel K-Means | 0.99 |
| Affinity Propagation | 0.37 |
| Latent Class Analysis | 0.67 |

### Supplementary table 6a

Cluster stability based on jaccard score of cluster membership overlap based on k-means from bootstrapped data run 100 times.

| Methods | AD error | UD error |
| --- | --- | --- |
| K-Means | 0.27 | 0.33 |
| Kernel K-Means | 0.13 | 0.27 |
| Affinity Propagation | 0.37 | 0.36 |
| Latent Class Analysis | 0.46 | 0.35 |

### Supplementary table 6b

Concordance between test set cluster membership determined by cluster method and cluster membership based on a decision tree trained on the original cluster results for each cluster method for the AD and UD test data sets.

| var | rsqr | ad_rsqr | predict |
| --- | --- | --- | --- |
| Memory | 0.008345186912 | 0.001159282469 | FALSE |
| Confusion | 0.008345186912 | 0.001159282469 | FALSE |
| Neuropsychological | 0.008345186912 | 0.001159282469 | FALSE |
| motor | 0.008345186912 | 0.001159282469 | FALSE |
| Anxiety comorbidity | 0.008345186912 | 0.001159282469 | FALSE |
| Atherosclerosis | 0.008345186912 | 0.001159282469 | FALSE |
| Atrial_fibrilation | 0.008345186912 | 0.001159282469 | FALSE |
| Cancer | 0.008345186912 | 0.001159282469 | FALSE |
| Depression_CM | 0.008345186912 | 0.001159282469 | FALSE |
| Diabetes | 0.008345186912 | 0.001159282469 | FALSE |
| Haemorrhagic_Stroke | 0.008345186912 | 0.001159282469 | FALSE |
| Hearing_Loss | 0.008345186912 | 0.001159282469 | FALSE |
| Heart_Failure | 0.008345186912 | 0.001159282469 | FALSE |
| Hypercholesterolemia | 0.008345186912 | 0.001159282469 | FALSE |
| Hyperglycemia | 0.008345186912 | 0.001159282469 | FALSE |
| Hypertension | 0.008345186912 | 0.001159282469 | FALSE |
| Kidney_disease | 0.008345186912 | 0.001159282469 | FALSE |
| Rhumatoid_Arthritis | 0.008345186912 | 0.001159282469 | FALSE |
| k means | 0 | 0 | FALSE |
| kernel k means | 0 | 0 | FALSE |
| affinity propagation | 0 | 0 | FALSE |
| Latent Class Analysis | 0 | 0 | FALSE |

### Supplementary table 7a

R squared values and adjusted r squared value for each variable based on a linear regression predicting decline in mmse score.

###

| hr | pval | lower_conf | upper_conf | names |
| --- | --- | --- | --- | --- |
| 0.5459139538 | 0 | 0.4986118653 | 0.5977034759 | Memory |
| 1.542066739 | 0 | 1.406779816 | 1.690363907 | Confusion |
| 1.325180167 | 3.21E-09 | 1.207243814 | 1.454637791 | neuropsychological |
| 1.012862076 | 0.9004408877 | 0.8290785421 | 1.237385282 | motor |
| 0.8595446312 | 0.006383625755 | 0.7709609106 | 0.958306657 | Anxiety_CM |
| 0.6661522676 | 0.0005569812584 | 0.5289263663 | 0.8389803796 | Atherosclerosis |
| 1.336411761 | 1.51E-06 | 1.187484623 | 1.504016439 | Atrial_fibrilation |
| 1.18153685 | 0.0004437181666 | 1.07652286 | 1.296794874 | Cancer |
| 0.9705816321 | 0.5500765994 | 0.8800432945 | 1.070434501 | Depression_CM |
| 0.835866775 | 0.007167009372 | 0.7334724396 | 0.9525555806 | Diabetes |
| 1.151332215 | 0.3607309995 | 0.8510582384 | 1.55755013 | Haemorrhagic_Stroke |
| 0.9932518579 | 0.8849459694 | 0.906211016 | 1.088652903 | Hearing_Loss |
| 1.845378944 | 0 | 1.614546587 | 2.109213495 | Heart_Failure |
| 6.14E-06 | 0.9782605061 | 0 | Inf | Hypercholesterolemia |
| 1.365365478 | 0.06787283146 | 0.9773848186 | 1.907358138 | Hyperglycemia |
| 0.9574835543 | 0.3360049677 | 0.8763793234 | 1.046093549 | Hypertension |
| 0.9170492223 | 0.07739248622 | 0.8330196814 | 1.00955511 | Kidney_disease |
| 1.055899914 | 0.7041083734 | 0.7974636294 | 1.398088374 | Rhumatoid_Arthritis |
| 1.846494381 | 0 | 1.624505847 | 2.098817623 | km2 |
| 0.9308953922 | 0.3229327664 | 0.8076727552 | 1.072917497 | km3 |
| 1.28364002 | 0.005106699751 | 1.077805682 | 1.528783647 | km4 |
| 1.138622374 | 0.07255705629 | 0.9881876054 | 1.31195828 | km5 |
| 1.473772293 | 0 | 1.350473553 | 1.608328253 | kk1 |
| 0.9971779518 | 0.9728479006 | 0.8474188114 | 1.173403109 | ap2 |
| 1.768648624 | 4.66E-15 | 1.533541696 | 2.039799742 | ap3 |
| 1.107035156 | 0.1862347958 | 0.9520919641 | 1.28719376 | ap4 |
| 1.71480554 | 1.35E-13 | 1.486574534 | 1.978076425 | ap5 |
| 1.072458555 | 0.5379043405 | 0.8584493482 | 1.339819703 | lca2 |
| 1.528066983 | 6.96E-05 | 1.239950809 | 1.883130111 | lca3 |
| 0.8926194956 | 0.2968978143 | 0.7210581039 | 1.105000498 | lca4 |
| 0.9041921517 | 0.3388213235 | 0.7355874391 | 1.111442914 | lca5 |
| 1.530314745 | 1.50E-05 | 1.262147859 | 1.855458695 | lca6 |

### Supplementary table 7b

results of the cox proportional hazard ratio time till death of each variable included in the cluster analysis and cluster membership

###

| hr | pval | lower_conf | upper_conf | names |
| --- | --- | --- | --- | --- |
| 0.7139461731 | 7.88E-15 | 0.6557682059 | 0.7772855308 | Memory |
| 1.556281001 | 0 | 1.433749692 | 1.689284097 | Confusion |
| 1.439713858 | 0 | 1.327035999 | 1.561959128 | neuropsychological |
| 1.137098767 | 0.1519657641 | 0.9538079785 | 1.355612067 | motor |
| 1.046292823 | 0.3321483485 | 0.9548472359 | 1.146496141 | Anxiety_CM |
| 0.7372051438 | 0.001674144377 | 0.6095484659 | 0.8915967384 | Atherosclerosis |
| 1.118026964 | 0.04973682961 | 1.000128301 | 1.24982394 | Atrial_fibrilation |
| 0.8939755624 | 0.01083207434 | 0.8201353912 | 0.9744638687 | Cancer |
| 1.093652738 | 0.03832131036 | 1.00482779 | 1.190329649 | Depression_CM |
| 0.8598397856 | 0.009521947394 | 0.7670798871 | 0.9638167672 | Diabetes |
| 1.154850936 | 0.2951918305 | 0.8819748902 | 1.512152669 | Haemorrhagic_Stroke |
| 0.957526476 | 0.2955242881 | 0.8827432114 | 1.038645147 | Hearing_Loss |
| 1.021923505 | 0.7789873901 | 0.878296128 | 1.189038202 | Heart_Failure |
| 1.017213033 | 0.9201277844 | 0.7286788993 | 1.419997691 | Hyperglycemia |
| 0.9248006511 | 0.04890721835 | 0.855576646 | 0.9996255138 | Hypertension |
| 0.9197497186 | 0.0550077479 | 0.8444233301 | 1.001795562 | Kidney_disease |
| 1.374578772 | 0.004829660164 | 1.101733649 | 1.714994184 | Rhumatoid_Arthritis |
| 1.520727001 | 1.30E-13 | 1.361055952 | 1.699129714 | km2 |
| 0.8952791431 | 0.06224790679 | 0.7969991618 | 1.005678277 | km3 |
| 0.9488428602 | 0.5189903425 | 0.8088798838 | 1.113024061 | km4 |
| 0.893038159 | 0.0663068343 | 0.7914667362 | 1.007644563 | km5 |
| 1.295926283 | 4.42E-11 | 1.199758339 | 1.399802674 | kk1 |
| 1.017394499 | 0.7934512304 | 0.8941840762 | 1.157582196 | ap2 |
| 1.209544535 | 0.002553890045 | 1.068920234 | 1.368668994 | ap3 |
| 0.8243135895 | 0.002623398912 | 0.7268302276 | 0.9348715395 | ap4 |
| 1.444421073 | 1.72E-09 | 1.281515591 | 1.628035002 | ap5 |
| 0.6569448957 | 2.02E-05 | 0.5415502156 | 0.7969281215 | lca2 |
| 1.049542349 | 0.5908165546 | 0.8799268245 | 1.251853122 | lca3 |
| 0.9401275704 | 0.4736936096 | 0.7940324486 | 1.113102935 | lca4 |
| 0.8620851043 | 0.07660371767 | 0.7314979736 | 1.01598467 | lca5 |
| 1.404855054 | 1.62E-05 | 1.203709498 | 1.639612985 | lca6 |

### Supplementary table 7c

cox proportional hazard ratio time till assisted living of each variable included in the cluster analysis and cluster membership

###

### Supplementary codelists

### Delusion

| Read code | Read Term | Medcode |
| --- | --- | --- |
| 1BH1.00 | Grandiose delusions | 32875 |
| 225F.00 | O/E - delusion of persecution | 52523 |
| Eu05200 | [X]Organic delusional [schizophrenia-like] disorder | 21986 |
| Eu22y00 | [X]Other persistent delusional disorders | 66077 |
| Eu22y11 | [X]Delusional dysmorphophobia | 40981 |
| 1BH..00 | Delusions | 1915 |
| ZR5..00 | Delusions-symptoms-states inventory | 54464 |
| 225E.00 | O/E - paranoid delusions | 12472 |
| E03y000 | Organic delusional syndrome | 10543 |
| Eu22000 | [X]Delusional disorder | 34389 |
| Eu22z00 | [X]Persistent delusional disorder, unspecified | 49223 |
| 1BH..11 | Delusion | 17982 |
| Eu45212 | [X]Dysmorphophobia nondelusional | 30680 |
| Eu24.00 | [X]Induced delusional disorder | 51302 |
| Eu22100 | [X]Delusional misidentification syndrome | 62405 |
| Eu2..00 | [X]Schizophrenia, schizotypal and delusional disorders | 17281 |
| 1BH0.00 | Delusion of persecution | 43462 |
| Eu22.00 | [X]Persistent delusional disorders | 28562 |
| Eu23300 | [X]Other acute predominantly delusional psychotic disorders | 44307 |

### Hallucination

| Read code | Read Term | Medcode |
| --- | --- | --- |
| E021.00 | Drug-induced paranoia or hallucinatory states | 45997 |
| Eu16500 | [X]Mental & behav dis due to hallucinogens: psychotic disord | 54983 |
| R001.00 | [D]Hallucinations | 2455 |
| E03y100 | Organic hallucinosis syndrome | 47109 |
| Ryu5300 | [X]Other hallucinations | 53761 |
| R001100 | [D]Hallucinations, gustatory | 53990 |
| 1B1e.00 | Hypnopompic hallucination | 85836 |
| Eu10511 | [X]Alcoholic hallucinosis | 6467 |
| R001000 | [D]Hallucinations, auditory | 12120 |
| F481K00 | Visual hallucinations | 15066 |
| Eu2y.11 | [X]Chronic hallucinatory psychosis | 31738 |
| R001300 | [D]Hallucinations, tactile | 64131 |
| 1B1E.00 | Hallucinations | 1914 |
| TJ96z00 | Adverse reaction to hallucinogen NOS | 64302 |
| 1B1b.00 | Transient hallucinations | 19518 |
| E013.00 | Alcohol withdrawal hallucinosis | 25110 |
| R001200 | [D]Hallucinations, olfactory | 25283 |
| R001z00 | [D]Hallucinations NOS | 19916 |
| Eu05000 | [X]Organic hallucinosis | 25338 |
| R001400 | [D]Visual hallucinations | 12064 |

### Agitation

| Read code | Read Term | Medcode |
| --- | --- | --- |
| 1B16.11 | Agitated - symptom | 5811 |
| 1B16.00 | Agitated | 3881 |
| E112.11 | Agitated depression | 5879 |
| E135.00 | Agitated depression | 1055 |
| J101y00 | Other specified oesophagitis | 35037 |
| J101200 | Chemical oesophagitis | 34836 |
| 2256 | O/E - agitated | 17086 |
| 1B1O.00 | Restless | 8123 |

### Aggression

| Read code | Read Term | Medcode |
| --- | --- | --- |
| Eu91112 | [X]Unsocialised aggressive disorder | 27794 |
| E2C0000 | Aggressive outburst | 2641 |
| Eu91111 | [X]Conduct disorder, solitary aggressive type | 33906 |
| E2C1.00 | Nonaggressive unsocial conduct disorder | 46452 |
| Eu60311 | [X]Aggressive personality disorder | 20839 |
| 1P5..00 | Aggressive behaviour | 10577 |
| BBGA.00 | [M]Aggressive fibromatosis | 55886 |
| E213.11 | Aggressive personality | 6339 |
| E2C0.00 | Aggressive unsocial conduct disorder | 2040 |
| E2C1z00 | Nonaggressive unsocial conduct disorder NOS | 20182 |
| E2C0z00 | Aggressive unsocial conduct disorder NOS | 24352 |
| J614200 | Chronic aggressive hepatitis | 1755 |
| E21y300 | Passive-aggressive personality disorder | 35763 |
| E293000 | Adjustment reaction with aggression | 6075 |

### Depression

| Read code | Read Term | Medcode |
| --- | --- | --- |
| 1B17.00 | Depressed | 1996 |
| 1B17.11 | C/O - feeling depressed | 4824 |
| 1B1U.00 | Symptoms of depression | 9796 |
| 1B1U.11 | Depressive symptoms | 10438 |
| 1BP..00 | Loss of interest | 30740 |
| 1BP0.00 | Loss of interest in previously enjoyable activity | 59869 |
| 1BQ..00 | Loss of capacity for enjoyment | 25435 |
| 1BT..00 | Depressed mood | 10015 |
| 1BT..11 | Low mood | 8928 |
| 1BU..00 | Loss of hope for the future | 53148 |

### Anxiety

| Read code | Read Term | Medcode |
| --- | --- | --- |
| 1466 | H/O: anxiety state | 3407 |
| E2D0.00 | Disturbance of anxiety and fearfulness childhood/adolescent | 31522 |
| E2D0000 | CHILDHOOD AND ADOLESCENT OVERANXIOUSNESS DISTURBANCE | 35619 |
| E2D0100 | CHILDHOOD AND ADOLESCENT FEARFULNESS DISTURBANCE | 56026 |
| E2D0z00 | Disturbance anxiety and fearfulness childhood/adolescent NOS | 35594 |
| Eu93200 | [X]Social anxiety disorder of childhood | 29907 |
| Eu93y12 | [X]CHILDHOOD OVERANXIOUS DISORDER | 61430 |
| 8G94.00 | Anxiety management training | 9125 |
| 8HHp.00 | Referral for guided self-help for anxiety | 28925 |
| Z4I7.00 | Acknowledging anxiety | 22159 |
| Z4I7100 | Recognising anxiety | 62935 |
| Z4I7211 | Reducing anxiety | 26295 |
| Z4L1.00 | Anxiety counselling | 7999 |
| E200200 | Generalised anxiety disorder | 4659 |
| E200400 | Chronic anxiety | 1758 |
| E200500 | Recurrent anxiety | 4634 |
| Eu41100 | [X]Generalized anxiety disorder | 10344 |
| Eu41111 | [X]Anxiety neurosis | 962 |
| E200300 | Anxiety with depression | 655 |
| Eu34114 | [X]Persistant anxiety depression | 15220 |
| Eu41200 | [X]Mixed anxiety and depressive disorder | 11913 |
| Eu41211 | [X]Mild anxiety depression | 7749 |
| E200100 | Panic disorder | 4069 |
| E200111 | Panic attack | 462 |
| E280.00 | Acute panic state due to acute stress reaction | 11940 |
| Eu41000 | [X]Panic disorder [episodic paroxysmal anxiety] | 8205 |
| Eu41011 | [X]Panic attack | 6408 |
| Eu41012 | [X]Panic state | 4081 |
| 1Bb1.00 | Fear of getting cancer | 18967 |
| E202B00 | Cancer phobia | 1510 |
| E262000 | CARDIAC NEUROSIS | 15292 |
| Eu45311 | [X]CARDIAC NEUROSIS | 44269 |
| Eu45313 | [X]GASTRIC NEUROSIS | 63259 |
| 285..00 | NEUROTIC CONDITION, INSIGHT PRESENT | 15811 |
| 286..00 | POOR INSIGHT INTO NEUROTIC CONDITION | 5274 |
| E200.00 | Anxiety states | 636 |
| E200000 | Anxiety state unspecified | 6939 |
| E200z00 | Anxiety state NOS | 4534 |
| Eu4..00 | [X]NEUROTIC, STRESS - RELATED AND SOMOFORM DISORDERS | 23808 |
| Eu41.00 | [X]Other anxiety disorders | 5385 |
| Eu41112 | [X]Anxiety reaction | 35825 |
| Eu41113 | [X]Anxiety state | 50191 |
| Eu41300 | [X]Other mixed anxiety disorders | 44321 |
| Eu41y00 | [X]Other specified anxiety disorders | 24066 |
| Eu41y11 | [X]Anxiety hysteria | 28167 |
| Eu41z00 | [X]Anxiety disorder, unspecified | 23838 |
| Eu41z11 | [X]Anxiety NOS | 25638 |
| Z4I7200 | Alleviating anxiety | 28381 |

### Apathy

| Read code | Read Term | Medcode |
| --- | --- | --- |
| R00z700 | [D]Demoralization and apathy | 18296 |
| 2254 | O/E - apathetic | 9435 |
| 1BP..00 | Loss of interest | 30740 |
| 1BP0.00 | Loss of interest in previously enjoyable activity | 59869 |

### Sleep

| Read code | Read Term | Medcode |
| --- | --- | --- |
| Fy03.00 | Sleep apnoea | 7603 |
| Fy03.11 | Obstructive sleep apnoea | 8148 |
| H5B..00 | Sleep apnoea | 23779 |
| H5B0.00 | Obstructive sleep apnoea | 20748 |
| R005100 | [D]Insomnia with sleep apnoea | 48539 |
| R005300 | [D]Hypersomnia with sleep apnoea | 36301 |
| R005311 | [D]Sleep apnoea syndrome | 2506 |
| R005312 | [D]Syndrome sleep apnoea | 20438 |
| C380200 | Extreme obesity with alveolar hypoventilation | 38059 |
| C38y.11 | Pickwickian syndrome | 24755 |
| C38y000 | Pickwickian syndrome | 38294 |
| Z1M..00 | Sleep and rest interventions | 22081 |
| R005800 | [D]Sleep dysfunction with sleep stage disturbance | 54458 |
| R005100 | [D]Insomnia with sleep apnoea | 48539 |
| Eu51z11 | [X]Emotional sleep disorder NOS | 21032 |
| 38D0.00 | Pittsburgh sleep quality index | 104942 |
| E274D11 | Restless sleep | 19514 |
| 8HTn.00 | Referral to sleep clinic | 95887 |
| 8Q0..00 | Sleep management | 12072 |
| Fy0..00 | Sleep disorders | 2329 |
| R005311 | [D]Sleep apnoea syndrome | 2506 |
| 9Nk0.00 | Seen in sleep clinic | 93615 |
| E274000 | Unspecified non-organic sleep disorder | 16434 |
| R005000 | [D]Sleep disturbance, unspecified | 1244 |
| E274C00 | Other sleep stage or arousal dysfunction | 55179 |
| ZV75312 | [V]Screening for sleeping sickness | 64498 |
| Fy00.00 | Disorders of initiating and maintaining sleep | 5921 |
| Fy02.00 | Disorders of the sleep-wake schedule | 8997 |
| R005.00 | [D]Sleep disturbances | 8084 |
| 1BX2.00 | Sleeping pattern | 60806 |
| R005z00 | [D]Sleep dysfunction NOS | 15407 |
| Fy03.00 | Sleep apnoea | 7603 |
| E274F00 | Inversion of sleep rhythm | 36745 |
| R005500 | [D]Sleep rhythm inversion | 16447 |
| R005.12 | [D]Sleep rhythm problems | 31236 |
| 1B1Q.00 | Poor sleep pattern | 7725 |
| 1BX9.00 | Light sleep | 60974 |
| Fyu5800 | [X]Other sleep disorders | 53912 |
| 1BX..00 | Sleep observations | 10989 |
| 1BX1.00 | Excessive sleep | 25211 |
| 7065800 | Sleep studies | 56809 |
| Eu51213 | [X]Psychogenic inversion of sleep rhythm | 101729 |
| Z1M1.00 | Disturbing sleep | 101913 |
| 7065A00 | Sleep studies NEC | 85670 |
| R005900 | [D]Sleep dysfunction with arousal disturbance | 41737 |
| 9b9Y.00 | Sleep studies - specialty | 104005 |
| 8G9B.00 | Sleep hygiene behaviour education | 96037 |
| Eu51000 | [X]Nonorganic insomnia | 30626 |
| 1B1B100 | Middle insomnia | 5675 |
| 1B1B200 | Late insomnia | 4597 |
| E274100 | Transient insomnia | 15515 |
| R005200 | [D]Insomnia NOS | 750 |
| E274200 | Persistent insomnia | 16115 |
| R005.11 | [D]Insomnia - symptom | 10349 |
| 1B1B.11 | C/O - insomnia | 4537 |
| 1B1B000 | Initial insomnia | 3523 |
| E274111 | Insomnia NOS | 4023 |
| E274.12 | Insomnia due to nonorganic sleep disorder | 26546 |
| 1B1B.00 | Cannot sleep - insomnia | 21305 |
| 38D1.00 | Insomnia severity index | 98268 |

### Eating

| 8HTN.00 | Referral to eating disorders clinic | 11612 |
| --- | --- | --- |
| R07z.11 | [D]Trouble eating | 2868 |
| ZR2U.00 | Binge eating scale | 22779 |
| Eu50411 | [X]Psychogenic overeating | 17439 |
| ZR3i.11 | CES - Compulsive eating scale | 94876 |
| E275y00 | Other specified non-organic eating disorder | 61236 |
| 67K8.00 | Cycle of change stage, healthy eating | 103471 |
| 9Nk9.00 | Seen in eating disorder clinic | 95883 |
| Eu50.00 | [X]Eating disorders | 6159 |
| 38Do.00 | Eating disorder examination questionnaire | 101131 |
| Eu50z00 | [X]Eating disorder, unspecified | 36946 |
| ZR3i.00 | Compulsive eating scale | 108910 |
| ZC2CD00 | Dietary advice for eating disorder | 67510 |
| ZRBS.11 | EBRS - Eating behaviour rating scale | 72383 |
| ZV4K300 | [V]Inappropriate diet and eating habits | 17643 |
| ZRBR.00 | Eating attitudes test | 68148 |
| Eu50y00 | [X]Other eating disorders | 34995 |
| ZR3W.00 | Clinical eating disorder rating instrument | 103029 |
| R036000 | [D]Excessive eating | 17642 |
| ZRBT.00 | Eating disorders inventory | 66331 |
| ZRhI.00 | Restrained eating inventory | 94654 |
| E275z00 | Non-organic eating disorder NOS | 32892 |
| ZRBA.00 | Diagnostic survey for eating disorder | 98836 |
| Z786100 | Eating practice | 11900 |
| E275.00 | Other and unspecified non-organic eating disorders | 7743 |
| Eu50400 | [X]Overeating associated with other psychological disturbncs | 39383 |
| ZRBA.11 | DSED - Diagnostic survey for eating disorders | 92686 |
| ZRh3.00 | Reasons for eating scale | 99111 |
| ZRBU.00 | Eating inventory | 64631 |
| 1FF..00 | Binge eating | 26518 |
| ZRBT.11 | EDI - Eating disorders inventory | 62365 |
| ZM1B400 | Does not prepare food for eating | 32785 |
| 1614 | Excessive eating - polyphagia | 35490 |
| ZRBR.11 | EAT - Eating attitudes test | 50141 |
| R030000 | [D]Appetite loss | 1855 |
| 1612.12 | Loss of appetite - symptom | 6607 |
| 1613 | Appetite increased | 16092 |
| Eu50y12 | [X]Psychogenic loss of appetite | 17203 |
| E275600 | Non-organic loss of appetite | 22820 |
| 1615 | Reduced appetite | 13081 |
| 161..00 | Appetite symptom | 6099 |
| 161Z.00 | Appetite symptom NOS | 14717 |

### Suspicion/paranoia

| Read code | Read Term | Medcode |
| --- | --- | --- |
| E002.00 | Senile dementia with depressive or paranoid features | 44674 |
| E103100 | Subchronic paranoid schizophrenia | 104760 |
| E12..00 | Paranoid states | 4261 |
| E021.00 | Drug-induced paranoia or hallucinatory states | 45997 |
| E133.00 | Acute paranoid reaction | 15053 |
| E210.00 | Paranoid personality disorder | 5652 |
| E103400 | Acute exacerbation of chronic paranoid schizophrenia | 53032 |
| E015.00 | Alcoholic paranoia | 30404 |
| Eu22y13 | [X]Paranoia querulans | 55236 |
| E134.00 | Psychogenic paranoid psychosis | 24345 |
| E103300 | Acute exacerbation of subchronic paranoid schizophrenia | 51322 |
| Eu22015 | [X]Paranoia | 4843 |
| Eu22012 | [X]Paranoid state | 11172 |
| E004200 | Arteriosclerotic dementia with paranoia | 55467 |
| Eu22300 | [X]Paranoid state in remission | 101720 |
| Eu05211 | [X]Paranoid organic state | 24009 |
| 225E.00 | O/E - paranoid delusions | 12472 |
| Eu22y12 | [X]Involutional paranoid state | 50248 |
| E123.00 | Shared paranoid disorder | 62680 |
| E103200 | Chronic paranoid schizophrenia | 31362 |
| Eu24.12 | [X]Induced paranoid disorder | 47230 |
| E103.00 | Paranoid schizophrenia | 1494 |
| Eu60014 | [X]Sensitive paranoid personality disorder | 48687 |
| E021z00 | Drug-induced paranoia or hallucinatory state NOS | 26481 |
| E002z00 | Senile dementia with depressive or paranoid features NOS | 41089 |
| Eu20000 | [X]Paranoid schizophrenia | 16764 |
| E12y000 | Paranoia querulans | 66766 |
| Eu02z16 | [X] Senile dementia, depressed or paranoid type | 27759 |
| Eu22011 | [X]Paranoid psychosis | 2113 |
| E103000 | Unspecified paranoid schizophrenia | 33383 |
| E103z00 | Paranoid schizophrenia NOS | 9281 |
| E021000 | Drug-induced paranoid state | 12628 |
| Eu60000 | [X]Paranoid personality disorder | 21338 |
| E001200 | Presenile dementia with paranoia | 30032 |
| E12y.00 | Other paranoid states | 31589 |
| Eu23312 | [X]Psychogenic paranoid psychosis | 27770 |
| 1BH3.00 | Paranoid ideation | 22643 |
| E002000 | Senile dementia with paranoia | 18386 |
| E121.00 | Chronic paranoid psychosis | 3890 |
| E12yz00 | Other paranoid states NOS | 31455 |
| Eu10513 | [X]Alcoholic paranoia | 30162 |
| E120.00 | Simple paranoid state | 14743 |
| E12z.00 | Paranoid psychosis NOS | 12771 |
| E103500 | Paranoid schizophrenia in remission | 36172 |

### Delirium

| Read code | Read Term | Medcode |
| --- | --- | --- |
| Eu04.00 | [X]Delirium, not induced by alcohol+other psychoactive subs | 25066 |
| E001100 | Presenile dementia with delirium | 49513 |
| E003.00 | Senile dementia with delirium | 37015 |
| Eu04y00 | [X]Other delirium | 52394 |
| Eu04000 | [X]Delirium not superimposed on dementia, so described | 68125 |
| Eu04100 | [X]Delirium superimposed on dementia | 53446 |
| E031.11 | Delirium - subacute organic | 24077 |
| E030.11 | Delirium - acute organic | 22466 |
| E010.12 | Delirium tremens | 1476 |
| E010.11 | DTs - delirium tremens | 22277 |
| Eu04z00 | [X]Delirium, unspecified | 53924 |
| E004100 | Arteriosclerotic dementia with delirium | 56912 |
| Eu10411 | [X]Delirium tremens, alcohol induced | 17259 |

### Mood disorders

| Read code | Read Term | Medcode |
| --- | --- | --- |
| E11..00 | Affective psychoses | 14656 |
| E11z.00 | Other and unspecified affective psychoses | 41992 |
| E11z000 | Unspecified affective psychoses NOS | 54607 |
| E11zz00 | Other affective psychosis NOS | 33425 |
| E211.00 | Affective personality disorder | 14979 |
| E211000 | Unspecified affective personality disorder | 16178 |
| E211z00 | Affective personality disorder NOS | 51497 |
| Eu3..00 | [X]Mood - affective disorders | 5726 |
| Eu34.00 | [X]Persistent mood affective disorders | 42857 |
| Eu34011 | [X]Affective personality disorder | 26839 |
| Eu34y00 | [X]Other persistent mood affective disorders | 50243 |
| Eu34z00 | [X]Persistent mood affective disorder, unspecified | 39767 |
| Eu3y.00 | [X]Other mood affective disorders | 28008 |
| Eu3y000 | [X]Other single mood affective disorders | 50998 |
| Eu3y011 | [X]Mixed affective episode | 30688 |
| Eu3y100 | [X]Other recurrent mood affective disorders | 29921 |
| Eu3y111 | [X]Recurrent brief depressive episodes | 19054 |
| Eu3yy00 | [X]Other specified mood affective disorders | 29579 |
| Eu3z.00 | [X]Unspecified mood affective disorder | 37090 |
| Eu3z.11 | [X]Affective psychosis NOS | 31633 |
| E292400 | Adjustment reaction with anxious mood | 56924 |
| 3880100 | Visual analogue mood scale | 106647 |
| ZRby.00 | Profile of mood states | 44287 |
| Z4I8.00 | Interpreting mood | 66333 |
| ZRaH.11 | MACL - Mood affective checklist | 64584 |
| 1BT..00 | Depressed mood | 10015 |
| 1S42.00 | Manic mood | 22713 |
| 1S40.00 | Dysphoric mood | 48349 |
| 1BS..00 | Normal mood symptoms | 54508 |
| ZRLfH00 | Health of the Nation Outcome Scale item 7 - depressed mood | 55288 |
| ZRby.11 | POMS - Profile of mood states | 100194 |
| ZR2G.00 | Behaviour and mood disturbance scale | 28442 |
| 1BT..12 | Sad mood | 26028 |
| 1BT..11 | Low mood | 8928 |
| ZRby100 | Profile of mood states, bipolar | 30282 |
| ZRLfI00 | Health of the Nation Outcome Scale item 7 - depressed mood | 96038 |
| Eu3yy00 | [X]Other specified mood affective disorders | 29579 |
| Eu34z00 | [X]Persistent mood affective disorder, unspecified | 39767 |
| Eu30212 | [X]Mania with mood-incongruent psychotic symptoms | 48632 |
| Eu30211 | [X]Mania with mood-congruent psychotic symptoms | 37102 |
| Eu3..00 | [X]Mood - affective disorders | 5726 |
| Eu34.00 | [X]Persistent mood affective disorders | 42857 |
| Eu3y000 | [X]Other single mood affective disorders | 50998 |
| Eu3y100 | [X]Other recurrent mood affective disorders | 29921 |
| 1BY..00 | Elevated mood | 18575 |
| 225K.00 | O/E - fearful mood | 26331 |
| 1BO..00 | Mood swings | 6021 |
| Eu3y.00 | [X]Other mood affective disorders | 28008 |
| ZRVZ.00 | Lindamood auditory conceptualisation test | 70775 |
| 1S41.00 | Ecstatic mood | 100826 |
| ZRaH.00 | Mood affective checklist | 34275 |
| 1S4..00 | Mood observations | 12416 |
| Eu3z.00 | [X]Unspecified mood affective disorder | 37090 |
| Eu34y00 | [X]Other persistent mood affective disorders | 50243 |
| E11z100 | Rebound mood swings | 3489 |
| Eu05300 | [X]Organic mood [affective] disorders | 24000 |
| R00z700 | [D] Demorilization and apathy | 18296 |

### Falls

| Read code | Read Term | Medcode |
| --- | --- | --- |
| U10H.00 | [X]Other fall from one level to another | 92721 |
| ZV71B00 | [V]Examination and observation following a fall | 6785 |
| U10H600 | [X]Other fall frm one level to anoth occ indust/constr area | 95961 |
| T170600 | MVTA-fall down bus stairs - pedal cyclist injured | 73257 |
| U10Hy00 | [X]Other fall frm one levl to anothr occ at oth specif plce | 72468 |
| TCyz.00 | Other accidental fall NOS | 11308 |
| TN70.00 | Injury ?accidental, fall from residential premises | 66371 |
| TN72.00 | Injury ?accidental, fall from natural site | 73097 |
| TC4yz00 | Other fall from one level to another NOS | 7876 |
| TC4..00 | Other fall from one level to another | 33887 |
| U10z600 | [X]Unspecified fall occurrn at industrial/construction area | 53455 |
| TC4y.00 | Other fall from one level to another | 41853 |
| U10zy00 | [X]Unspecified fall, occurrence at other specified place | 96546 |
| T170100 | MVTA-fall down bus stairs - motor vehicle passenger injured | 38564 |
| T170y00 | MVTA-fall down bus stairs - other specified person injured | 71496 |
| TN7z.00 | Injury ?accidental, fall from high place NOS | 69499 |
| TN7..00 | Injury ?accidental, fall from high place | 20668 |
| U10zz00 | [X]Unspecified fall, occurrence at unspecified place | 61170 |
| U10Hz00 | [X]Othr fall frm one level to anothr occurrn at unspec plce | 49233 |
| U10H500 | [X]Other fall frm one level to anothr occ at trde/serv area | 68604 |
| TCy..00 | Other falls | 8730 |
| TC...00 | Accidental falls | 6815 |
| 615B.11 | IUD fallen out | 22945 |
| TCz..00 | Accidental falls NOS | 6835 |
| 16D1.00 | Recurrent falls | 8694 |
| T183600 | MVTA-fall from moving MV - pedal cyclist injured | 53412 |
| T253.00 | MVNTA - fall from moving motor vehicle, except off-road MV | 39013 |
| U10z.00 | [X]Unspecified fall | 24776 |
| U10J600 | [X]Other fall on same levl, occurrn at indust/constuct area | 68608 |
| U10H300 | [X]Othr fall from one level to anothr occ sport/athlet area | 67229 |
| U10H200 | [X]Othr fall frm one level to anothr, sch inst/pub adm area | 68609 |
| U10z000 | [X]Unspecified fall, occurrence at home | 10419 |
| U10H400 | [X]Othr fall from one level to anothr occurrn street/h'way | 68562 |
| U10H000 | [X]Other fall from one level to another, occurrence at home | 51669 |
| T171.00 | MVTA - fall from car in street while boarding/alighting | 41114 |
| T171700 | MVTA-fall from car in street - pedestrian injured | 38497 |
| R200.12 | [D] Geriatric fall | 4859 |
| U131.00 | [X]Drowning and submersion following fall into bath-tub | 92100 |
| T189100 | MVTA-obj falling on mov MV - motor vehicle passenger injured | 85219 |
| U10J000 | [X]Other fall on same level, occurrence at home | 43191 |
| U10J100 | [X]Other fall on same level, occurrnce in resident instit'n | 72474 |
| U10z100 | [X]Unspecified fall, occurrence in residential institution | 46303 |
| U10z400 | [X]Unspecified fall, occurrence on street and highway | 97327 |
| U10J.00 | [X]Other fall on same level | 48496 |
| T183.00 | MVTA - fall from motor vehicle while in motion | 12597 |
| T170400 | MVTA-fall down bus stairs - occupant of tram injured | 85212 |
| T170.00 | MVTA - fall down stairs of motor bus while board/alighting | 18097 |

### Orientation

| Read code | Read Term | Medcode |
| --- | --- | --- |
| Z7CC.00 | Observations related to orientation | 89325 |
| Z7A1600 | Orientation training | 69258 |
| Z7A1711 | RO - Reality orientation | 101365 |
| Z7CC111 | Orientation good | 61005 |
| Z7CC700 | Spatial disorientation | 55460 |
| Z7CC.11 | Orientation observations | 46919 |
| Z7A1700 | Reality orientation | 51351 |
| ZRhm.00 | Samaritan orientation test | 93909 |
| Z7CC600 | Disorientation for person | 66012 |
| R00zX00 | [D]Disorientation, unspecified | 20683 |
| Z7CC312 | Orientation poor | 64219 |
| Z7CC311 | Orientation confused | 19719 |
| ZRVU.00 | Life orientation test | 62751 |
| ZRkB.00 | Short orientation - memory - concentration test | 24819 |
| ZRVU.11 | LOT - Life orientation test | 103254 |
| Ryu5700 | [X]Disorientation, unspecified | 52811 |
| F481J00 | Visual disorientation syndrome | 31572 |
| Z7CC200 | Spatial orientation good | 97963 |
| ZRJ..11 | GOAT - Galvestone orientation and amnesia test | 106429 |
| Z7CMC11 | Visual disorientation syndrome | 44820 |

### Difficulty walking

| Read code | Read Term | Medcode |
| --- | --- | --- |
| 398C.00 | Deterioration in ability to walk up stairs | 107068 |
| 3993 | Difficulty walking up stairs | 55875 |
| ZOA7700 | Unable to walk up step | 66091 |
| ZOA7A00 | Difficulty walking up step | 59521 |
| Z6A2.00 | Walking with patient - mobilisation | 36621 |
| N097200 | Walking difficulty due to lower leg | 36256 |
| 13C4.00 | Needs walking aid in home | 13128 |
| N097400 | Walking difficulty due to other specified site | 63199 |
| N097.00 | Difficulty in walking | 6375 |
| 39BZ.00 | Other walking aid | 46659 |
| 8D44100 | Walking tripod | 43815 |
| N097300 | Walking difficulty due to ankle and foot | 41492 |
| 33P..00 | Timed six metre walk test | 102945 |
| 39D1.00 | Walks in 1 minute 30-59 metres | 46417 |
| 39D..00 | Walking distance | 17591 |
| 3983 | Independent walking | 36994 |
| ZOA7F00 | Difficulty walking down step | 63322 |
| N097000 | Walking difficulty due to unspecified site | 2537 |
| 2999 | Toe-walking gait | 24993 |
| ZOA6800 | Does not walk up stairs | 57096 |
| 398B.00 | Deterioration in ability to walk | 103686 |
| ZOA7C00 | Unable to walk down step | 97495 |
| 33B9600 | 6-minute walk test | 100593 |
| Ryu3100 | [X]Difficulty in walking, not elsewhere classified | 95777 |
| ZOA6900 | Difficulty walking up stairs | 46866 |
| ZOA7E00 | Does not walk down step | 70317 |
| ZOA6E00 | Difficulty walking down stairs | 88777 |
| ZOA7900 | Does not walk up step | 60785 |
| ZOA6B00 | Unable to walk down stairs | 39498 |
| 39B..00 | Walking aid use | 9990 |

### Memory

| Read code | Read Term | Medcode |
| --- | --- | --- |
| Z7CEH13 | Bad memory | 26434 |
| Z7CEH12 | Memory deficit | 50418 |
| Z7CEH11 | Memory dysfunction | 39915 |
| Z7CEH15 | Poor memory | 12583 |
| Z7CEH14 | Memory problem | 12057 |
| E2A1000 | Mild memory disturbance | 6387 |
| Z7CE414 | Memory disturbance | 105538 |
| ZRLK.00 | Graham-Kendal memory for designs test | 106868 |
| ZRrO.11 | WMS - Wechsler memory scale | 44956 |
| ZRh6.00 | Recognition memory test | 96881 |
| Z7CEC11 | Loss of memory for recent events | 9786 |
| R00z011 | [D]Memory deficit | 7711 |
| 3A90.00 | Memory: count down successful | 52791 |
| Z7CGI00 | Verbal memory | 22349 |
| 3A3..00 | Memory: present place | 53012 |
| Z7CA100 | Isolated memory skills | 66172 |
| 3A20.00 | Memory: present time not known | 53146 |
| 3A9..00 | Memory: count down | 52781 |
| Z7CE114 | No problem with memory | 50816 |
| Z7CEN11 | Invents experiences to compensate for loss of memory | 64892 |
| 3A11.00 | Memory: own age known | 52784 |
| 1S21.00 | Disturbance of memory for order of events | 67163 |
| Z7CGK00 | Verbal memory recall | 94985 |
| 1B1a.00 | Poor auditory sequential memory | 40821 |
| 1S2..00 | Memory observations | 19224 |
| 3A81.00 | Memory: important person known | 52790 |
| Z7CGA00 | Procedural memory | 62581 |
| 8HTY.00 | Referral to memory clinic | 22802 |
| Z7CE.11 | Observations relating to memory | 47480 |
| 7P10400 | Neuropsychology test of memory | 88787 |
| Z7CFO11 | Long-term memory loss | 47581 |
| Z7CF811 | Short-term memory loss | 10571 |
| Z7CEL00 | Mild memory disturbance | 51724 |
| Z7CFx00 | Memory aided by use of labels | 98287 |
| Z7CGH00 | Autobiographical memory | 50486 |
| Z7CFO00 | Poor long-term memory | 53978 |
| 8IEn.00 | Referral to memory clinic declined | 106295 |
| ZR1n.00 | Autobiographical memory interview | 61830 |
| ZRhS100 | Rivermead behavioural memory test - adult version | 65409 |
| Z7CEA13 | Impairment of primary memory | 65696 |
| Z7CEA11 | Impairment of working memory | 32367 |
| Z7CGF00 | Auditory memory | 56388 |
| Z7CEK00 | Minor memory lapses | 53507 |
| 3A6..00 | Memory: present month | 91077 |
| ZRBg.00 | Everyday memory questionnaire | 106998 |
| Z7A1500 | Memory retraining | 65365 |
| Z7CF800 | Poor short-term memory | 11410 |
| 3A80.00 | Memory: import.person not knwn | 52801 |
| 1B1A100 | Short-term memory loss | 103453 |
| 1B1Y.00 | Poor visual sequential memory | 39507 |
| 3A50.00 | Memory: own DOB not known | 53125 |
| 1B1A.00 | Memory loss - amnesia | 1993 |
| Z7CGJ00 | Verbal memory encoding | 43410 |
| Z7CEJ00 | Memory lapses | 19073 |
| Z7CGC00 | Visual memory | 104864 |
| Z7CE611 | Memory loss | 10123 |
| Z7CE612 | Memory gone | 68230 |
| Z7CE615 | Loss of memory | 19297 |
| Z7CE616 | LOM - Loss of memory | 12277 |
| 3A41.00 | Memory: present year known | 52787 |
| Z7CEC12 | No memory for recent events | 67802 |
| 3AA1.00 | Memory: address recall unsucc. | 53016 |
| ZRrh.00 | Williams memory assessment scales | 104472 |
| ZRF..00 | Fuld object memory evaluation | 98411 |
| Z7CFz00 | Memory aided by use of lists | 67951 |
| Z7A1300 | Memory skills training | 49265 |
| 8IE5000 | Initial memory assessment declined | 107353 |
| ZRkB.00 | Short orientation - memory - concentration test | 24819 |
| 3A91.00 | Memory: count down unsuccess. | 52805 |
| 3A7..00 | Memory: important event | 53015 |
| Z7CEB12 | Poor memory for remote events | 37191 |
| ZRqa.00 | Valentine auditory memory test | 64392 |
| 3A30.00 | Memory: present place not knwn | 53014 |
| Z7CE400 | Memory disturbance (& amnesia (& symptom)) | 51379 |
| 3A61.00 | Memory: present month known | 52789 |
| Z7CGL00 | Verbal memory for names | 46986 |
| Z7CF100 | Memory recall normal | 62972 |
| 3A4..00 | Memory: present year | 91078 |
| 1B1A.13 | Memory disturbance | 2908 |
| 1B1A.12 | Memory loss symptom | 5777 |
| 3A5..00 | Memory: own DOB | 52824 |
| ZRrg.00 | Wechsler memory scale revised | 66927 |
| Z7CFN12 | No problems with long-term memory | 47147 |
| 3A2..00 | Memory: present time | 52799 |
| 3A51.00 | Memory: own DOB known | 52788 |
| Z7CGP00 | Delayed verbal memory | 98798 |
| 3A40.00 | Memory: present year not known | 52948 |
| ZRhS.00 | Rivermead behavioural memory test | 38025 |
| 3AA..00 | Memory: address recall | 95076 |
| 3A10.00 | Memory: own age not known | 52947 |
| Z7CEF00 | Temporary loss of memory | 67998 |
| Z7CEH00 | Memory impairment | 10514 |
| ZR2X.11 | Memory concentration test | 36658 |
| 3A70.00 | Memory: important event not kn | 52800 |
| ZRkC.00 | Short-term memory test | 28704 |
| Z7CE415 | Loss of memory | 102880 |
| Z7CE412 | Memory loss symptom | 67838 |
| Z7CF111 | Global memory recall within normal limits | 63996 |
| Z7CE115 | Global memory recall within normal limits | 73290 |
| Z7CE113 | Memory intact | 59244 |
| Z7CF700 | Short-term memory within normal limits | 19382 |
| ZRrO.00 | Wechsler memory scale | 72737 |
| E2A1100 | Organic memory impairment | 6061 |
| 3A31.00 | Memory: present place known | 52786 |
| 3A60.00 | Memory: present month not knwn | 52825 |
| 3A8..00 | Memory: important person | 52826 |
| Z7CEM00 | Distortion of memory | 51739 |
| Z7CFw00 | Memory aided by use of diary | 59830 |
| 38C1500 | Initial memory assessment | 107351 |
| ZRh6.11 | RMT - Recognition memory test | 93236 |
| 3A21.00 | Memory: present time known | 52785 |
| 3A...11 | Memory assessment | 18274 |
| ZR2X.13 | Information-memory-concentration test | 64813 |
| ZRhS.11 | RBMT - Rivermead behavioural memory test | 73514 |
| 9Nk1.00 | Seen in memory clinic | 94164 |
| Z7CF.00 | Observations of memory performance | 66686 |
| ZR1n.12 | AMI - Autobiographical memory interview | 64660 |
| ZD11300 | Auditory memory therapy | 101356 |

### Language

| Read code | Read Term | Medcode |
| --- | --- | --- |
| ZS7B700 | Restricted language development | 54141 |
| ZT...00 | Speech and language observations | 10413 |
| Eu80y00 | [X]Other developmental disorders of speech and language | 46054 |
| ZS84.00 | Expressive language disorder | 32305 |
| ZS7..00 | Language impairment | 40065 |
| ZLEL200 | Discharge from hospital speech and language therapy service | 25483 |
| 13Z6Z00 | Language NOS | 24353 |
| ZS...00 | Speech and language disorder | 22896 |
| ZS81.00 | Developmental expressive language disorder | 45472 |
| ZR3A.11 | CELF - Carrow elicited language inventory | 26188 |
| ZL87211 | Referral to hospital speech and language therapist | 40623 |
| 13wW.00 | Main spoken language Wolof | 97039 |
| ZRqH.00 | Test of language competence | 68129 |
| ZD15.00 | Cognitive neuropsychological language therapy | 48526 |
| ZS7B100 | Expressive language delay | 12372 |
| ZLDP200 | Discharge by hospital-based speech and language therapist | 60449 |
| ZS7C600 | Language disorder associated with thought disorder | 32831 |
| ZR3X.00 | Clinical evaluation of language function revised | 96174 |
| 13ZA.00 | Language difficulty | 2807 |
| ZL87100 | Referral to community-based speech and language therapist | 32613 |
| ZT47200 | Unable to use language | 92228 |
| ZD...00 | Speech and language therapy | 18470 |
| 9NNj200 | Under care of speech and language therapist | 106399 |
| ZL4C111 | Under care of community speech and language therapist | 7443 |
| ZL4C200 | Under care of hospital-based speech and language therapist | 8374 |
| ZT4J400 | Does not use the elements of language | 102899 |
| 1Bc..00 | Speech and language observations | 104268 |
| ZT31.00 | Observations of use and content of language | 94143 |
| ZRh5.00 | Receptive expressive emergent language scale | 102101 |
| ZS5..00 | Speech and language dyspraxias | 43892 |
| ZD3..00 | Speech and language therapy regimes | 12226 |
| ZL4C100 | Under care of community-based speech and language therapist | 32742 |
| 9N0Q.00 | Seen in speech and language clinic | 2309 |
| ZL4C211 | Under care of hospital speech and language therapist | 25559 |
| ZRbf.00 | Psycholinguistic assessments of language process in aphasia | 94851 |
| ZD38200 | Cognitive behavioural language therapy | 22045 |
| 7P10300 | Neuropsychology test of language | 94197 |
| ZLDP100 | Discharge by community-based speech and language therapist | 51451 |
| ZRVI.00 | Language assessment remediation and screening procedure | 72784 |
| Eu80z00 | [X]Developmental disorder of speech and language unspecified | 6514 |
| ZD16.00 | Neurolinguistics language therapy | 42916 |
| E2F3.00 | Speech or language developmental disorder | 3567 |
| ZLEL.00 | Discharge from speech and language therapy service | 26190 |
| ZS72.00 | Receptive language impairment | 32542 |
| ZL4C.00 | Under care of speech and language therapist | 12027 |
| ZLDP111 | Discharge by community speech and language therapist | 25581 |
| ZL87200 | Referral to hospital-based speech and language therapist | 30278 |
| ZT32.00 | General observations of language and communication | 63704 |
| ZS6..00 | Speech and phonology impairments | 28845 |
| ZT...00 | Speech and language observations | 10413 |
| ZS67100 | Developmental speech disorder | 25574 |
| Ryu6000 | [X]Other and unspecified speech disturbances | 95670 |
| 8E21.00 | Speech therapy | 402 |
| ZD4K.00 | Speech exercises | 106883 |
| 9N29.00 | Seen by speech therapist | 25804 |
| 2B4..00 | O/E - speech defect | 24491 |
| ZT4F400 | Does not use speech appropriately | 57263 |
| 1B94.00 | Speech limited | 25709 |
| ZL4C111 | Under care of community speech and language therapist | 7443 |
| 1B9Z.00 | Speech problem NOS | 33403 |
| ZT23.00 | Speech problem | 32923 |
| R045.00 | [D]Other speech disturbance | 18039 |
| ZS4..00 | Motor speech disorder | 63935 |
| ZL87.11 | Refer to speech therapist | 11805 |
| ZS5..00 | Speech and language dyspraxias | 43892 |
| ZD3..00 | Speech and language therapy regimes | 12226 |
| E2F3z00 | Speech or language developmental disorder NOS | 1277 |
| 03J6.00 | Speech therapist | 4076 |
| ZV40100 | [V]Problems with communication, including speech | 20922 |
| ZT21.00 | Observations of quality of speech | 57938 |
| ZT22.00 | Observation of fluency of speech | 92307 |
| ZV57300 | [V]Speech therapy | 1238 |
| 1B9..11 | Speech problem - symptom | 9713 |
| ZT21300 | Incoherent speech | 32250 |
| ZT2..00 | Speech observations | 46982 |
| ZD...11 | Speech therapy | 90774 |
| ZV40111 | [V]Problems with speech | 6495 |
| ZT4E511 | Difficulty initiating speech | 51734 |
| Eu80z00 | [X]Developmental disorder of speech and language unspecified | 6514 |
| ZT22100 | Lack of fluency in speech | 62197 |
| ZS67.00 | Speech impairment | 18452 |
| ZT21112 | Observation of clarity of speech | 67999 |
| ZT21111 | Observation of intelligibility of speech | 102503 |
| 1B93.00 | Has difficulty with speech | 41125 |
| 1B9..00 | Speech problem | 2107 |
| 2B4Z.00 | O/E - speech defect NOS | 21035 |
| R045z00 | [D]Other speech disturbance NOS | 5688 |
| ZL4C.00 | Under care of speech and language therapist | 12027 |
| ZLDP111 | Discharge by community speech and language therapist | 25581 |
| Ryu6.00 | [X]Symptoms and signs involving speech and voice | 95732 |
| ZL87200 | Referral to hospital-based speech and language therapist | 30278 |
| ZT21211 | Unintelligible speech | 61809 |
| ZRhz.00 | Screening for developmental apraxia of speech | 94621 |

### Confusion

| Read code | Read Term | Medcode |
| --- | --- | --- |
| E030z00 | Acute confusional state NOS | 41537 |
| E030.00 | Acute confusional state | 4033 |
| E030400 | Acute confusional state, of cerebrovascular origin | 25114 |
| 2232 | O/E - mentally confused | 4874 |
| E030.12 | Toxic confusional state | 3486 |
| E031000 | Subacute confusional state, post traumatic | 61238 |
| 2841.11 | Confusion | 3991 |
| 2232.11 | O/E - confused | 16968 |
| E030200 | Acute confusional state, of endocrine origin | 94079 |
| E042.00 | Chronic confusional state | 4951 |
| 38DN.00 | Confusion, respiratory rate, BP, 65 year age or older score | 98746 |
| E031400 | Subacute confusional state, of cerebrovascular origin | 24035 |
| E030300 | Acute confusional state, of metabolic origin | 70409 |
| Z7CC311 | Orientation confused | 19719 |
| E030000 | Acute confusional state, post traumatic | 25051 |
| E031300 | Subacute confusional state, of metabolic origin | 38671 |
| 38DS.00 | Confusion, urea, respiratory rate, BP, 65 age or older score | 100133 |
| E132.00 | Reactive confusion | 7332 |
| R009.00 | [D]Confusion | 5188 |
| E031.00 | Subacute confusional state | 17021 |
| E030100 | Acute confusional state, of infective origin | 50683 |
| Eu44y13 | [X]Psychogenic confusion | 16988 |
| R009000 | [D]Toxic confusional state | 26104 |
| R009.11 | [D] Senile confusion | 3325 |
| E031z00 | Subacute confusional state NOS | 55784 |
| Eu04.12 | [X]Acute / subacute confusional state, nonalcoholic | 7389 |
| E031100 | Subacute confusional state, of infective origin | 69359 |
| 2841 | Confused | 1713 |

### Incontinence

| Read code | Read Term | Medcode |
| --- | --- | --- |
| 39H0.00 | Continence reassessment | 49417 |
| R083.00 | [D]Incontinence of urine | 3283 |
| 9Nl8.00 | Seen by continence nurse | 94673 |
| 8D74.11 | Catheter in situ | 8163 |
| 7B42100 | Insertion of bulbar urethral prosthesis | 23915 |
| 19E3.00 | Incontinent of faeces | 3381 |
| R083100 | [D]Urethral sphincter incontinence | 31220 |
| Z915.11 | Urinary catheter care | 17783 |
| Z1S2100 | Planned voiding two hourly | 57613 |
| Z1S2.00 | Planned voiding | 51015 |
| 7B31200 | Colposuspension of bladder neck | 4202 |
| 8D75.00 | Penile sheath provision | 24289 |
| 222K.00 | Smells of urine | 17829 |
| Kyu5A00 | [X]Other specified urinary incontinence | 52763 |
| 19E3.11 | Incontinent of faeces symptom | 6083 |
| 1A27.11 | Pis en deux | 108397 |
| 1A24.11 | Stress incontinence - symptom | 5844 |
| Z1S..00 | Toileting regimes | 39873 |
| 1A25.00 | Urgency | 5959 |
| 394..11 | Bladder-incontinence assessmnt | 13424 |
| 679H.11 | Promotion of continence | 29040 |
| R083z00 | [D]Incontinence of urine NOS | 15400 |
| Z1J2.00 | Attaching penile sheath | 46491 |
| 1593 | H/O: stress incontinence | 15918 |
| 3930 | Bowels: incontinent | 13426 |
| 8D74.00 | Indwelling urethral catheter | 34048 |
| R086200 | [D] Urgency of micturition | 8028 |
| 394..12 | Bladder- continence assessment | 13423 |
| R076.00 | [D]Incontinence of faeces | 1437 |
| 1A26.00 | Urge incontinence of urine | 3887 |
| 1A24.00 | Stress incontinence | 1929 |
| ZL62400 | Referral to continence nurse | 25901 |
| 1A36.00 | Terminal dribbling of urine | 2756 |
| 8C14.00 | Incontinence care | 2739 |
| 1A25.11 | Urgency of micturition | 583 |
| 3941 | Bladder: occasional accident | 13422 |
| 3940 | Bladder: incontinent | 13421 |
| Z89A100 | Does not perform toileting activities | 60557 |
| 39H..00 | Continence assessment | 40789 |
| 679H.00 | Health education - continence | 29039 |
| R076z00 | [D]Incontinence of faeces NOS | 15555 |
| 8HTX.00 | Referral to incontinence clinic | 25899 |
| 3931 | Bowels: occasional accident | 13429 |
| K198.00 | Stress incontinence | 3182 |
| K586.00 | Stress incontinence - female | 17620 |
| 8E97000 | Bladder drill | 33268 |
| 1A37.00 | Dribbling of urine | 5705 |
| 7B33800 | Insertion retropubic device stress urinary incontinence NEC | 98767 |
| 8C14.11 | Continence care | 12138 |
| 7D17.11 | Colporrhaphy and amputation of cervix uteri | 21695 |
| Z1J..00 | Procedures to aid continence | 46614 |
| 8156.11 | Urinary catheter care | 39099 |
| 8D7..00 | Urinary bladder control | 30981 |
| R076100 | [D]Sphincter ani incontinence | 27623 |
| 8HR6.00 | Refer to Urodynamic studies | 18998 |
| Z121400 | Assisting with toileting | 60929 |
| ZQ3C.00 | Bowels incontinence assessment | 43222 |
| 8H7w.00 | Referral to continence nurse | 29192 |
| Z89AE00 | Unable to use urine bottle | 58629 |
| 8D71.00 | Incontinence control | 48601 |
| 1A23.00 | Incontinence of urine | 6161 |
| Z89A200 | Difficulty performing toileting activities | 37563 |
| R083200 | [D] Urge incontinence | 17320 |
| Z1S1.00 | Regular toileting | 93070 |
| 7B42112 | Insertion of prosthesis for compression bulb of male urethra | 67850 |
| 16F..00 | Double incontinence | 5196 |
| 3973 | Difficulty performing toileting activities | 105224 |
| 7B31211 | Burch colposuspension | 17771 |
| ZLD7400 | Discharge by continence nurse | 43931 |
| ZL22400 | Under care of continence nurse | 45492 |
| 8D7..12 | Incontinence control | 17637 |
| 8D7..11 | Bladder control | 16592 |
| 8D7Z.00 | Urinary bladder control NOS | 47963 |
| 7B33C00 | Insertion retropubic dev fem stress urinary incontinence NEC | 94021 |
| 393..12 | Bowels-incontinence assessment | 31256 |
| 393..11 | Bowels - continence | 13428 |
| ZLA2400 | Seen by continence nurse | 22095 |
| 7B42113 | Insertion of Rosen prosthesis for male incontinence | 106791 |
| 7B42111 | Insertion of Kaufman prosthesis for male incontinence | 58675 |
| Z915.00 | Urinary catheter appliance procedures | 26051 |
| ZRas400 | OPCS continence disability scale | 101647 |
| Z89A.00 | Ability to perform toileting activities | 39811 |
| 8E97.00 | Bladder training | 9020 |
| 1A27.00 | Urge to pass urine again shortly after finishing voiding | 93952 |

### Seizures

| Read code | Read Term | Medcode |
| --- | --- | --- |
| Q480.12 | Seizures in newborn | 13304 |
| F25z.11 | Fit (in known epileptic) NOS | 3607 |
| 2824.11 | O/E - Jacksonian fit | 39530 |
| R003011 | [D]Pyrexial convulsion | 16792 |
| 2824 | O/E - focal (Jacksonian) fit | 57277 |
| R003.00 | [D]Convulsions | 1137 |
| 282..00 | O/E - fit/convulsion | 32662 |
| F254500 | Complex partial epileptic seizure | 11394 |
| Q480.11 | Fits in newborn | 21839 |
| R003z11 | [D]Seizure NOS | 1306 |
| F256z00 | Infantile spasms NOS | 49322 |
| Ryu7100 | [X]Other and unspecified convulsions | 72608 |
| F251y00 | Other specified generalised convulsive epilepsy | 45927 |
| 2822 | O/E - grand mal fit | 7811 |
| F251600 | Grand mal seizure | 5668 |
| F255600 | Simple partial epileptic seizure | 40105 |
| F25X.00 | Status epilepticus | 6271 |
| 1B64.00 | Had a convulsion | 9085 |
| F25H.00 | Generalised seizure | 106571 |
| F253.11 | Status epilepticus | 4093 |
| Fyu5900 | [X]Status epilepticus | 71801 |
| 1B63.11 | Fit - had one | 3652 |
| Fyu5200 | [X]Other status epilepticus | 59120 |
| F256.00 | Infantile spasms | 4478 |
| 2825 | O/E - psychomotor fit | 51517 |
| F250300 | Epileptic seizures - akinetic | 31830 |
| F250011 | Epileptic absences | 1715 |
| F251300 | Epileptic seizures - myoclonic | 4801 |
| 1B6B.00 | Febrile convulsion | 12662 |
| F251400 | Epileptic seizures - tonic | 5152 |
| 1B64.11 | Convulsion - symptom | 7808 |
| R003z00 | [D]Convulsion NOS | 15077 |
| F253.00 | Grand mal status | 5117 |
| F250200 | Epileptic seizures - atonic | 24309 |
| 1B63.00 | Had a fit | 1902 |
| F256.11 | Lightning spasms | 68486 |
| F132z12 | Myoclonic seizure | 8487 |
| R003200 | [D]Fit | 11025 |
| R003y00 | [D]Other specified convulsion | 25865 |
| R003100 | [D]Convulsions | 27647 |
| F251200 | Epileptic seizures - clonic | 18471 |
| R003211 | [D]Fit (in non epileptic) NOS | 6721 |
| F25y300 | Complex partial status epilepticus | 25330 |
| F252.00 | Petit mal status | 9886 |
| 282Z.00 | O/E - fit/convulsion NOS | 38457 |
| R003400 | [D]Nocturnal seizure | 99834 |
| 2827 | O/E - febrile convulsion | 6063 |
| 2823 | O/E - petit mal fit | 7809 |
| R003000 | [D]Convulsions | 614 |
| 1B27.00 | Seizures in response to acute event | 11505 |
| 2828 | Absence seizure | 8097 |
| 2824.12 | O/E - focal fit | 12098 |
| 282..11 | O/E - a convulsion | 6072 |
| 282..12 | O/E - a fit | 17136 |
| 282..13 | O/E - a seizure | 7275 |
| Q480.00 | Convulsions in newborn | 20005 |

### Fainting

| Read code | Read Term | Medcode |
| --- | --- | --- |
| 147B.00 | H/O: vasovagal faint | 11112 |
| 1B68.00 | Felt faint | 163 |
| R002100 | [D]Fainting | 184 |
| 1B6..11 | Faint symptom | 6201 |
| 1B62.00 | Syncope/vasovagal faint | 16267 |

### 
